# The effect of spin polarization on double electron–electron resonance (DEER) spectroscopy

**DOI:** 10.5194/mr-3-101-2022

**Published:** 2022-07-01

**Authors:** Sarah R. Sweger, Vasyl P. Denysenkov, Lutz Maibaum, Thomas F. Prisner, Stefan Stoll

**Affiliations:** 1 Department of Chemistry, University of Washington, Seattle, WA 98195, USA; 2 Institute of Physical and Theoretical Chemistry and Center of Biomolecular Magnetic Resonance, Goethe University Frankfurt am Main, Frankfurt, Germany

## Abstract

Double electron–electron resonance (DEER) spectroscopy measures the distribution of distances between two electron spins in the nanometer range, often on doubly spin-labeled proteins, via the modulation of a refocused spin echo by the dipolar interaction between the spins. DEER is commonly conducted under conditions where the polarization of the spins is small. Here, we examine the DEER signal under conditions of high spin polarization, thermally obtainable at low temperatures and high magnetic fields, and show that the signal acquires a polarization-dependent out-of-phase component both for the intramolecular and intermolecular contributions. For the latter, this corresponds to a phase shift of the spin echo that is linear in the pump pulse position. We derive a compact analytical form of this phase shift and show experimental measurements using monoradical and biradical nitroxides at several fields and temperatures. The effect highlights a novel aspect of the fundamental spin physics underlying DEER spectroscopy.

## 1 Introduction

Double electron–electron resonance (DEER) spectroscopy is a pulse electron paramagnetic resonance (EPR) technique utilized for determining distances between spin centers on a nanometer scale [Bibr bib1.bibx27]. The technique has seen use with studies of organic polymers but is most commonly used for structural studies in large biomolecules, such as proteins and nucleic acids [Bibr bib1.bibx30]. DEER resolves the full distribution of distances in an ensemble of proteins, making it possible to directly quantify protein conformational ensembles [Bibr bib1.bibx19].

DEER measures the amplitude of a spin echo as a function of the position of one or more pump pulses. The magnetic dipole–dipole coupling between spins leads to a modulation of the echo amplitude whose frequency is dependent on the magnitude of the coupling. The majority of DEER experiments are conducted on samples of dilute and uniformly distributed spin pairs on doubly labeled molecules or complexes. These samples give rise to an overall signal that is a product of an oscillatory intramolecular signal and an exponentially decaying intermolecular signal (Fig. [Fig Ch1.F1]a). The latter is often referred to as the background signal.

The behavior of the spins depends upon the temperature and magnetic field, which together determine the magnitude of thermal polarization (Fig. [Fig Ch1.F1]b). DEER spectroscopy using nitroxide radicals is often conducted at 50–70 K and 0.3–1.2 T. Under these experimental conditions, the Zeeman interactions are smaller than the thermal energy, and consequently the thermal spin polarization is small. Larger-than-thermal polarization can be generated utilizing photo-excited molecular triplet states [Bibr bib1.bibx11] or optical pumping in NV centers, which have a triplet ground state [Bibr bib1.bibx38]. Non-thermal spin polarizations are known to lead to out-of-phase electron spin echo amplitude modulation (OOP-ESEEM) in spin-correlated radical pairs [Bibr bib1.bibx29].

This work considers DEER signals under significant thermal spin polarization as obtainable at low temperatures and high magnetic fields. Expanding on previous work [Bibr bib1.bibx23], we show theoretically that the out-of-phase components of both the intramolecular and intermolecular signals are polarization-dependent. In particular, we derive a closed-form analytical expression for the intermolecular signal that reveals a polarization-dependent phase factor. We demonstrate this experimentally with monoradical and biradical nitroxide samples at several fields and temperatures.

**Figure 1 Ch1.F1:**
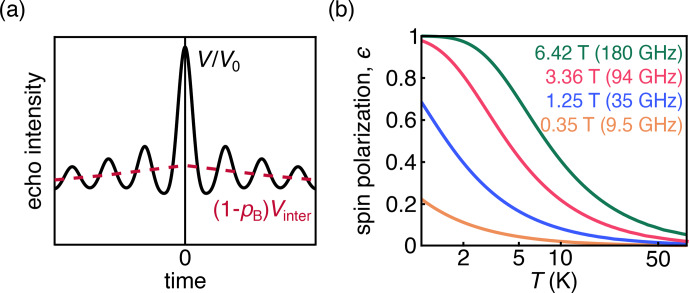
**(a)** DEER signal (black) with the oscillatory intramolecular signal; the contribution from the exponentially decaying intermolecular signal is shown in pink. **(b)** Dependence of thermal spin polarization ϵ on magnetic field and temperature.

The paper is structured as follows. Section [Sec Ch1.S2] steps through the analytical spin dynamics model showing the polarization-dependent echo phase, beginning from a two-spin system and then expanding to a uniform spatial distribution of spins. Section [Sec Ch1.S3] describes experimental details, and Sect. [Sec Ch1.S4] presents and discusses the experimental results. Section [Sec Ch1.S5] concludes.

## 2 Analytical model

In this section, we derive the analytical form of the intermolecular DEER signal, without the usual assumption of the high-temperature limit. The derivation within the high-temperature limit and the initial analytical expression for the intramolecular DEER signal of an isolated spin pair beyond the high-temperature limit has previously been reported [Bibr bib1.bibx26] and is derived in Sect. S1 in the Supplement. Section S1 in the Supplement derives the three-pulse DEER signal, the result of which is identical to the four-pulse DEER model, and thus the rest of the discussion will refer to the four-pulse DEER model to remain consistent with the experimental results.

The analysis is based on the standard model for four-pulse DEER of a frozen dilute solution of doubly spin-labeled molecules. We define two spectrally non-overlapping and separately addressable subsets of electron spins, A and B. (The sample also contains spins not affected by any pulses due to the broad EPR spectrum, particularly at high fields; we denote them as C spins.) The A spins are manipulated via pulses at the probe frequency, $\omega_{A}$ , while the B spins are inverted via a pulse at the pump frequency, $\omega_{B}$ . DEER measures the amplitude V(t) of the refocused electron spin echo as a function of the position in time of the pump pulse, t . It is given by

1 $$V(t)=V_{0}\cdot V_{\mathrm{intra}}(t)\cdot V_{\mathrm{inter}}(t).$$

The echo amplitude is a product of an overall amplitude, $V_{0}$ , a modulation function due to the intramolecular spin pairs, $V_{\mathrm{intra}}(t)$ , and an intermolecular modulation function, $V_{\mathrm{inter}}(t)$ , containing the contributions from spin pairs with spins on different molecules.

For an individual pair of one A spin located at position $r_{A}$ and one B spin located at $r_{B}$ (not necessarily on the same molecule), the DEER signal is [Bibr bib1.bibx23]

2 $$V_{\mathrm{AB}}(r,t)=(1-p_{B})+p_{B}\left(\mathrm{cos}(\omega t)+i\epsilon \;\sin(\omega t)\right).$$

Here, $r=r_{B}-r_{A}$ is the inter-spin distance vector, and ω is the dipolar coupling frequency

3 $$\omega (r)=D\frac{1-3\cos^{2}\theta_{\mathrm{AB}}}{r^{3}}\;r=|r|\;\mathrm{cos}\theta_{\mathrm{AB}}=r\cdot z/r$$

with the dipolar constant

4 $$D=\frac{\mu_{0}}{4\pi }\frac{\mu_{B}^{2}g_{e}^{2}}{\hbar }\approx 2\pi \cdot 52.04\;\mathrm{MHz}\;{\mathrm{nm}}^{3},$$

where $\mu_{0}$ is the magnetic constant, $\mu_{B}$ is the Bohr magneton, $g_{e}$ is g value of the free electron, ℏ is the reduced Planck constant, and z is the unit vector along the direction of the applied magnetic field. For systems with g values differing from $g_{e}$ , the respective g values can be used instead [Bibr bib1.bibx1]. In Eq. ([Disp-formula Ch1.E2]), $p_{B}$ is the inversion efficiency of the pump pulse, and ϵ is the polarization of the B spins, ranging between 0 and 1. For a spin- 1/2 , the polarization at thermal equilibrium is

5 $$\epsilon (B,T)=\frac{N_{\beta }-N_{\alpha }}{N_{\beta }+N_{\alpha }}=\tanh\frac{g_{e}\mu_{B}B}{2k_{B}T},$$

where $N_{\beta }$ and $N_{\alpha }$ are the populations of the ground and excited states, respectively, B is the magnetic field strength, $k_{B}$ is the Boltzmann constant, and T is the temperature. The dependence of the thermal polarization on B and T is shown in Fig. [Fig Ch1.F1]b. Equation ([Disp-formula Ch1.E2]) is derived under the assumption that the dipolar coupling frequency is small compared to the difference in resonance frequencies between the two spins. Equation ([Disp-formula Ch1.E2]) is the standard in-phase DEER signal with an additional polarization-dependent out-of-phase sin⁡ term that is proportional to ϵ and therefore significant at low temperatures and high magnetic fields or in the presence of spin hyperpolarization.

**Figure 2 Ch1.F2:**
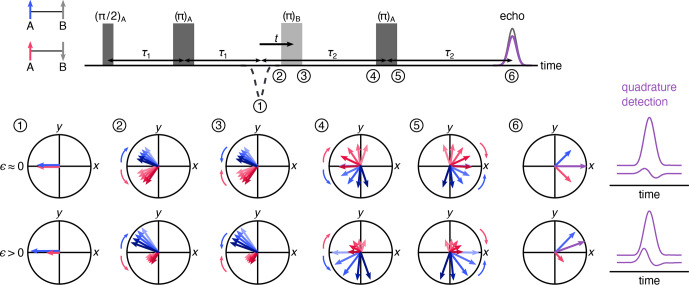
Vector model of the spin movement along the four-pulse DEER sequence. Cases of low- and high-polarization values are shown where the numbers indicate the vector picture at a given point in time during the pulse sequence. The vectors indicate the two sub-populations of A spins where those next to up B spins are colored blue and those next to down B spins are pink. The length of the arrows indicates the population. The overall magnetization is a sum of the two sub-populations, shown in purple. The respective echoes for low and high spin polarization values are shown on the right.

Figure [Fig Ch1.F2] uses a vector model in the rotating frame to visualize the dynamics of A spins through the four-pulse DEER sequence, illustrating how the polarization affects the echo phase. The vectors indicate the two sub-ensembles of A spins, one where the neighboring B spin is in the $m_{S}=+1/2$ state (blue) and the other where it is in the $m_{S}=-1/2$ state (pink). The lengths of the arrows indicate the respective populations. The first two pulses of the sequence form an A-spin echo at time point 1, with both A-spin sub-ensembles refocused with -x phase. As time passes, the spins precess and de-phase due to a distribution of precession frequencies. This distribution is indicated by a set of arrows in varying shades, darker indicating higher frequency. The mean precession frequencies of the two sub-ensembles differ by the dipolar coupling frequency ω . The pump pulse flips the B spins. This leaves all A-spin phases unaffected but shifts the frequencies of the two A-spin sub-ensembles in opposite directions by ±ω , so that the two sub-ensembles now accumulate phase in reversed directions while continuing to de-phase as before the pulse. The final π pulse on the A spins rotates both sub-ensembles around the y axis, and after evolution for $\tau_{2}$ , they refocus to give two echoes with opposite phases. The measured echo is proportional to the vector sum of these two sub-echoes. In the high-temperature regime ( ϵ≈0 , top), the populations of the two sub-ensembles are equal, and therefore the echo has a pure x phase (in-phase), with the quadrature component ( y phase) integrating to zero. In the presence of significant polarization ( ϵ>0 ), the two sub-echoes have significantly different amplitudes, and the overall echo phase is rotated from x , yielding a non-zero quadrature (out-of-phase) component.

The average of $V_{\mathrm{AB}}$ over a uniform orientational distribution of A–B spin pairs with fixed r is

6 $$\begin{array}{rl} V_{\mathrm{intra}}(r,t) & =\frac{F_{C}(z)\cos(\phi )+F_{S}(z)\sin(\phi )}{z}\\ & +i\;\epsilon \frac{F_{C}(z)\sin(\phi )-F_{S}(z)\cos(\phi )}{z}, \end{array}$$

with $\phi =Dt/r^{3}$ and $z=\sqrt{6\phi /\pi }$ . $F_{C}$ and $F_{S}$ are the Fresnel cosine and sine integral functions, respectively, which for imaginary arguments ( z is imaginary for t<0 ) have the properties $F_{C}(ai)=i\;F_{C}(a)$ and $F_{S}(ai)=-i\;F_{S}(a)$ . The in-phase (real) and out-of-phase (imaginary) components of this signal are shown in Fig. [Fig Ch1.F3]a.

For a uniform, random, spatial distribution of B spins in a sample, the signal is a product of all individual $V_{\mathrm{AB}}$ signals starting from Eq. ([Disp-formula Ch1.E2]), additionally averaged over all B-spin configurations:

7 $$V_{\mathrm{inter}}(t)=\langle \prod_{b=1}^{N_{B}}V_{\mathrm{AB}}(r_{b},t)\rangle .$$

Here, $N_{B}$ is the number of B spins in a configuration, and $r_{b}$ is the vector from the A spin to the b th B spin. To arrive at the product form, the dipolar couplings among B spins are neglected. A somewhat involved derivation (spelled out in Sect. S2) shows that Eq. ([Disp-formula Ch1.E7]) evaluates to

8 $$V_{\mathrm{inter}}(t)=\exp(-k|t|)\cdot \exp(i\alpha \epsilon kt),$$

where

9 $$k=\frac{8\pi^{2}}{9\sqrt{3}}p_{B}c_{B}D\;\alpha =\frac{\sqrt{3}+\ln\left(2-\sqrt{3}\right)}{\pi }\approx 0.13213.$$

The first factor in Eq. ([Disp-formula Ch1.E8]) is an exponential decay function, as has been derived and observed before. The decay rate constant k depends on the B-spin concentration $c_{B}$ and on the inversion efficiency $p_{B}$ . The second factor is an additional hitherto unappreciated phase factor with a phase that grows linearly with t and is proportional to the spin polarization ϵ . This phase factor leads to a non-zero out-of-phase signal for t≠0 as long as the spin polarization ϵ is large enough. The signal is plotted in Fig. [Fig Ch1.F3]b. Equation ([Disp-formula Ch1.E8]) was also confirmed numerically using Monte Carlo simulations (Fig. S1 in the Supplement).

**Figure 3 Ch1.F3:**
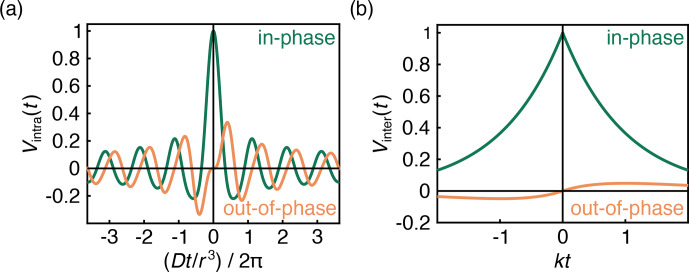
**(a)** The theoretical powder-averaged polarized DEER signal for a single A–B spin pair (Eq. [Disp-formula Ch1.E6]). **(b)** The theoretical DEER time trace for an A spin coupled to a uniform three-dimensional distribution of B spins at full spin polarization (Eq. [Disp-formula Ch1.E8], ϵ=1 ).

The extrema in the out-of-phase part of $V_{\mathrm{inter}}(t)$ are located at

10 $$t=\pm t_{\mathrm{OOP}}=\pm \frac{1}{k}\cdot \frac{\arctan(\alpha \epsilon )}{\alpha \epsilon }\approx \pm \frac{1}{k}.$$

At these points, the magnitude of $V_{\mathrm{inter}}$ has decayed to $e^{-1}\approx 0.368$ . These time points depend only on concentration and inversion efficiency, not on polarization. Numerically, these quantities are related via

11 $$p_{B}\cdot \left(c_{B}/\mathrm{mM}\right)\cdot (t_{\mathrm{OOP}}/\mu s)\approx 1.$$

At $t=t_{\mathrm{OOP}}$ , the out-of-phase amplitude of the intermolecular signal is

12 ImVintertOOP≈e-1αϵ1+(αϵ)2≈e-1αϵ≈0.0486ϵ.

At high temperatures and low fields, ϵ≈0 , and the out-of-phase component vanishes. The slope around t=0 is dictated by the degree of polarization. Even at full polarization, the amplitude of the out-of-phase component is small (Fig. [Fig Ch1.F3]b), in contrast to that of an isolated A–B pair (Fig. [Fig Ch1.F3]a), where the out-of-phase signal reaches ≈0.335ϵ at $(Dt/r^{3})/2\pi \approx 0.403$ .

The Fourier transform of Eq. ([Disp-formula Ch1.E8]) is a Lorentzian lineshape

13 $$\mathrm{FT}(V_{\mathrm{inter}}(t),\omega )=\sqrt{\frac{2}{\pi }}\frac{k}{k^{2}+(\omega +\alpha \epsilon k{)}^{2}}$$

centered at angular frequency ω=-αϵk and a full width at half maximum of fwhm=2k . The frequency ω is proportional to the polarization, the spin concentration, and the inversion efficiency. For $c_{B}=1\;\mathrm{mM}$ , $p_{B}=1$ , and ϵ=1 , this gives a frequency of ω/2π≈-0.021 MHz and a line width of fwhm/2π≈0.317 MHz.

## 3 Materials and methods

### 3.1 Q-band DEER

2,2,6,6-Tetramethylpiperidine-1-oxyl (TEMPO) was obtained from Sigma-Aldrich and used to create an approximately 1 mM solution in 50:50 (w:w) D ${}_{2}$ O and d ${}_{8}$ -glycerol. For measurements at Q-band, 30–50 µL of sample was syringed into 1.50 mm outer diameter (O.D,) and 1.1 mm inner diameter (I.D.) quartz tubes (Sutter Instrument). Q-band experimental data were measured on a Bruker Elexsys E580 spectrometer equipped with a Bruker D2 dielectric resonator at 33.9 GHz, 1.21 T, at 11 and 40 K. The microwave power was amplified with a 390 W Applied Systems Engineering TWT amplifier. For both temperatures, the measurements were conducted at the field value that gave the maximum echo amplitude at the pump frequency. Optimal pulse lengths were determined using a Rabi nutation experiment. For the 11 K data, a standard four-pulse DEER sequence was used with rectangular observer pulses at 33.842 GHz, the π/2 and π pulses being 22 and 44 ns and a sech/tanh pump pulse applied at 33.922 GHz with a 200 ns length and 80 MHz bandwidth. For the 40 K data, rectangular observer pulses with 22 and 44 ns lengths were applied at 33.828 GHz, and a sech/tanh pump pulse was applied at 33.908 GHz with a 200 ns length and 80 MHz bandwidth. Experiments at both temperatures utilized a 64-step phase cycle [Bibr bib1.bibx37]. The values for $\tau_{1}$ and $\tau_{2}$ were 4000 and 4200 ns, respectively, for the 11 K data and 3000 and 3200 ns for the 40 K data. To keep the sample at thermal equilibrium for each echo, the spectrum was collected with one shot per point and a 1 s shot repetition time (SRT) at 11 K. At 40 K, the data were collected with 10 shots per point and a 3 ms repetition time.

### 3.2 G-band DEER

4-Hydroxy-2,2,6,6-tetramethylpiperidine-1-oxyl (TEMPOL) was obtained from Sigma-Aldrich Chemie GmbH and used to create an approximately 1.0 mM solution with 45:55 (v/v) D ${}_{2}$ O : d ${}_{8}$ -glycerol. The homo-biradical nitroxide was synthesized according to the procedure previously published [Bibr bib1.bibx3], and 0.16 mg was dissolved in 0.61 mL of deuterated toluene, giving a final concentration of 0.25 mM, corresponding to 0.5 mM total spin concentration. The solution was placed in a 0.55 mm O.D. 0.4 mm I.D. quartz sample capillary that resulted in 250 nL of sample volume inside the TE011 cylindrical cavity resonator. Samples were frozen after insertion into the cryostat. The G-band experiment were performed on a home-built pulse EPR spectrometer operating at 180 GHz, 6.42 T, and temperatures of 5, 40, and 50 K [Bibr bib1.bibx28]. For the monoradical experiments, a standard four-pulse DEER sequence with rectangular pulses was used with the observer π/2 and π pulses being 36 and 58 ns, respectively, while the pump pulse was 58 ns with a frequency offset of +50 MHz. The values for $\tau_{1}$ and $\tau_{2}$ were 4000 and 5000 ns, respectively. Each DEER trace has 80 points and was recorded with 10 shots per point with 500 ms SRT for 5 K and 100 shots per point with 6 ms SRT for 40 K. This repetition time value was chosen as a 4-fold longer time with respect to the $T_{1}$ value for nitroxide radicals in deuterated glycerol / D ${}_{2}$ O solution at 6.4 T and 40 K. For the experiments at 5 K, the repetition time was chosen according to the saturation recovery experiment (Fig. S5). Each trace was phased individually. For the biradical experiments, a standard four-pulse DEER sequence with rectangular pulses was used with the observer π/2 and π pulses being 48 and 90 ns, respectively, while the pump pulse was 80 ns with a frequency offset of +80 MHz. The values for $\tau_{1}$ and $\tau_{2}$ were 500 and 5000 ns, respectively. Each DEER trace has 80 points and was recorded with 10 shots per point with repetition times of 6 ms at 50 K and 500 ms at 5 K.

## 4 Results and discussion

To experimentally observe the polarization-dependent phase of the DEER signal, we performed DEER experiments on a 1 mM TEMPO sample in D ${}_{2}$ O : d ${}_{8}$ -glycerol at several magnetic fields and temperatures. The results are shown in Fig. [Fig Ch1.F4]. To fit the model from Eq. ([Disp-formula Ch1.E8]) to these data, we extended the model to

14 $$V(t)=V_{0}\cdot \exp\left(-k|t-t_{0}|\right)\cdot \exp\left(i\alpha \epsilon q_{B}k\left(t-t_{0}\right)\right),$$

where the fit parameters are the overall signal amplitude $V_{0}$ , the decay rate constant k , the zero-time shift $t_{0}$ , and an additional phenomenological fit factor $q_{B}$ discussed below. ϵ was calculated from the temperature and magnetic field using Eq. ([Disp-formula Ch1.E5]) and is given in Fig. [Fig Ch1.F4].

**Figure 4 Ch1.F4:**
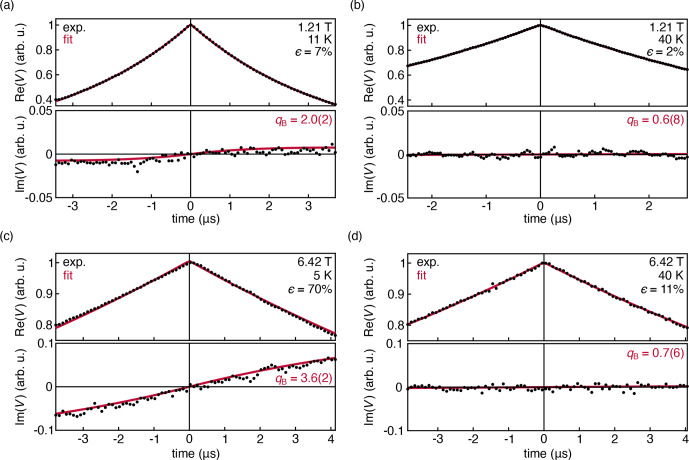
Experimental DEER traces for a 1.0 mM solution of TEMPO in 50:50 D ${}_{2}$ O : d ${}_{8}$ -glycerol, measured at 33.9 GHz at 11 K **(a)** and 40 K **(b)**. Experimental DEER traces for a 1.0 mM solution of TEMPOL in 45:55 D ${}_{2}$ O : d ${}_{8}$ -glycerol, measured at 180 GHz and 6.42 T at 5 K **(c)** and 40 K **(d)**. The data were fit with Eq. ([Disp-formula Ch1.E14]) and are shown with their 95 % confidence interval in parentheses. All fit parameters are shown in Table S1 in the Supplement.

Figure [Fig Ch1.F4]a shows the DEER decay measured at 33.9 GHz, 1.21 T, and 11 K, where the thermal polarization is about 7 %. The out-of-phase signal is small but observable. The shape of the observed out-of-phase signal is reproduced by the fit. However, it grows with t , more quickly than predicted by theory. This is indicated by the fitted value $q_{B}\approx 2.0(2)$ , whereas we expect $q_{B}=1$ from theory (Eq. ([Disp-formula Ch1.E8]). When the temperature is raised to 40 K, Fig. [Fig Ch1.F4]b, thermal polarization is reduced significantly to 2 %, and the out-of-phase signal flattens and is not observable as expected.

Figure [Fig Ch1.F4]c and d show the DEER decays measured at 180 GHz and 6.42 T at 5 and 40 K, where the thermal polarization is 70 % and 11 %, respectively. Again, the model of Eq. ([Disp-formula Ch1.E14]) was fit to the data. In the 5 K data, the out-of-phase signal is now clearly visible, and the experiment again confirms the shape predicted by the model, with a discrepancy in the phase rotation rate ( $q_{B}\approx 3.6(2)$ ). There is also a very slight asymmetry in the in-phase signal that is not captured by the model. At 40 K, the amplitude of the out-of-phase signal is negligible and the in-phase signal is symmetric, consistent with the theoretical prediction.

**Figure 5 Ch1.F5:**
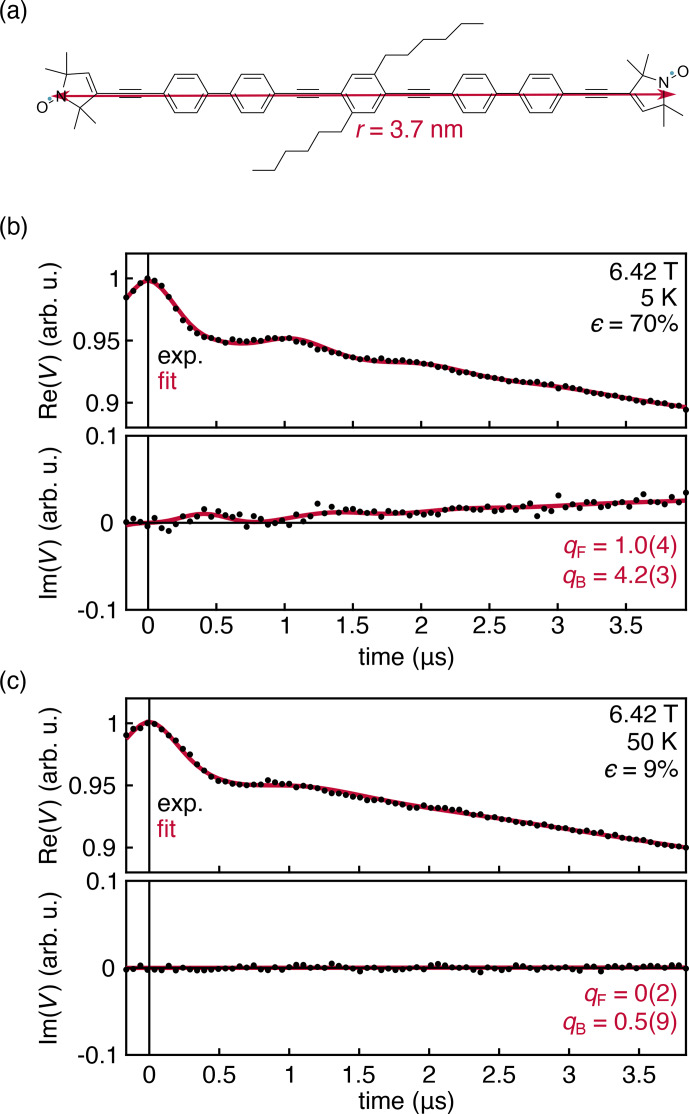
**(a)** Nitroxide biradical with an inter-spin distance of 3.7 nm [Bibr bib1.bibx31]. Experimental DEER traces for a 0.25 mM solution of the biradical in deuterated toluene, measured at 180 GHz and 6.42 T at 5 **(b)** and 50 K **(c)**. The data were fit with Eqs. ([Disp-formula Ch1.E14])–([Disp-formula Ch1.E16]). Fit parameters are shown with their 95 % confidence interval. All fit parameters are listed in Table S2.

The experimental results in Fig. [Fig Ch1.F4] were obtained with a monoradical solution. To further investigate the origins of the observed deviation of the intermolecular contribution from the theoretical prediction, we measured a frozen solution of the nitroxide biradical shown in Fig. [Fig Ch1.F5]a at 180 GHz, 6.42 T, and 5 and 50 K. Under these conditions, the thermal polarization is 70 % and 9 %, respectively. The results are shown in Fig. [Fig Ch1.F5]. The intermolecular signal was fit according to Eq. ([Disp-formula Ch1.E14]), and the intramolecular signal was fit using an adaptation of Eq. ([Disp-formula Ch1.E6]) that incorporates an additional phenomenological factor $q_{F}$ , analogous to $q_{B}$ in Eq. ([Disp-formula Ch1.E14]).

15 $$\begin{array}{rl} V_{\mathrm{AB}}(r,t) & =\frac{F_{C}(z)\cos(\phi )+F_{S}(z)\sin(\phi )}{z}\\ & +i\epsilon q_{F}\frac{F_{C}(z)\sin(\phi )-F_{S}(z)\cos(\phi )}{z} \end{array}$$

This was added to verify experimental temperature and to test whether the observed discrepancy in the intermolecular background signals ( $q_{B}\neq 1$ ) is also present in the intramolecular signal. The intra-biradical distance distribution was modeled as a Gaussian distribution

16 $$P(r)=\frac{1}{w\sqrt{2\pi }}\exp\left(-\frac{{\left(r-r_{0}\right)}^{2}}{2w^{2}}\right),$$

with the peak position $r_{0}$ and the standard deviation w as fit parameters.

The experimental data are shown in Fig. [Fig Ch1.F5]. The dipolar oscillations are clearly resolved, and the theory once again matches the overall shape of the experimental data with a discrepancy for the intermolecular signal captured by the fit factor $q_{B}=4.2(3)$ . However, this discrepancy does not appear for the intramolecular signal, where the fit factor of $q_{F}=1.0(4)$ confirms both that the sample temperature is 5 K ± 0.1 K and that the theoretical expression in Eq. ([Disp-formula Ch1.E6]) is correct. As the temperature is raised to 40 K and the polarization value drops significantly, the out-of-phase component disappears, consistent with theory.

Together, the experiments reveal that the theory predicts the out-of-phase component of the intramolecular signal quantitatively but underpredicts it for the intermolecular contribution. Experiments also show a very slight polarization-dependent asymmetry in the intermolecular signal that is not captured by theory.

A range of experimental and theoretical factors was taken into consideration to potentially explain the origin of the observed discrepancy, the results of which are provided in the SI. Many instrumental factors can be excluded based on control experiments and on the data from the biradical. Pump–probe pulse excitation band overlap for the experimental conditions used (observer frequency 180 000 GHz, pump frequency 180 050 GHz, pulse widths 40–80 ns) is no more than approximately 2.7 % (Fig. S3). This overlap is too small to create significant shifts in the data. Gain imbalance between in-phase and out-of-phase detectors was determined to be insignificant, as the experiment run with the detector phase rotated by 90 ${}^{\circ }$ yielded no visible changes in the data (Fig. S4). Experiments were run with detection both on and off the echo to verify that the observed signal arises from the echo and not the background of the resonator (Fig. S5). The detector phase was shown to drift slowly over the course of the experiments. This was accounted for by running the traces with as short of an acquisition time as possible and phasing each trace individually. However, it appears that this is not the origin of the observed discrepancy as traces with backwards and forward sweeps of t give approximately the same result (Fig. S6). Temperature drifts were found to be insignificant, as an temperature sensor near the resonator indicated temperature stability within ±0.1 K during the experiment. The fact that theory fits the out-of-phase component of the intramolecular signal in Fig. [Fig Ch1.F5]b well excludes many potential instrumental origins of the discrepancy between theory and the intermolecular signal.

Samples were run at sufficient cryoprotectant levels to eliminate aggregation as a source of error, at least for the experiments utilizing a monoradical sample [Bibr bib1.bibx6]. For all 180 GHz data, samples were not frozen rapidly outside the spectrometer but rather after being placed into the resonator. For the biradical data, field sweeps of a sample frozen outside and one frozen in the resonator showed little difference (Fig. S8), further indicating that aggregation is not likely a major source of error.

Possible saturation effects were tested by running a saturation recovery experiment showing that a 500 ms repetition time is sufficiently slow so as to not saturate the A spins (Fig. S7). One interesting possibility could be the occurrence of selective saturation. If the saturation is selective with respect to the A-spin sub-populations, then, if the less populated A-spin sub-population is saturated more, the echo phase shift would increase.

The possibility of spectral diffusion of the A spins can be excluded from consideration as the cause of the discrepancy, as it would decrease the overall signal equally in both the in-phase and out-of-phase signals. Spectral diffusion of the B spins is expected to have no effect on DEER. One possibility is that there is selective spectral diffusion, affecting the two sub-populations of A spins differently, though this is unlikely given the excitation profiles of the pulses shown in Fig. S3.

The discrepancy may be due to flip–flop terms of the A–B or B–B spin coupling Hamiltonians that are neglected in the current theory. Inclusion of the A–B flip–flop term has been found to be important for the correct description of DEER signals from Gd(III)–Gd(III) spin pairs with short distances [Bibr bib1.bibx9]. For a spin concentration c=0.1 mM $=6\cdot 10^{16}$ cm ${}^{-3}$ (obtained from 1 mM total concentration and 10 % excitation), the modal nearest-neighbor distance between spins is about 13 nm (Fig. S2), which corresponds to a dipolar coupling frequency of less than 0.05 MHz. The flip–flop term only matters if the dipolar coupling frequency between two adjacent spins is on the same order as or larger than their resonance frequency difference Δω . Since A and B spins are frequency-separated by at least 50 MHz (see Materials and methods), the A–B flip–flop terms are negligible. In addition, the accurate model fit of the intramolecular signal in Fig. [Fig Ch1.F5]b (with $q_{F}=1$ ) confirms that A–B flip–flop terms are irrelevant. Since in the G-band experiments the excitation bandwidth of the pump pulse is about $1.7/t_{p}\approx 30$ MHz, two adjacent B spins have a typical Δω on the order of a few megahertz. Therefore, the B–B flip–flop terms are likely of minor relevance, although it is not certain that they are negligible.

It is possible that demagnetization effects play a role in the observed discrepancy. In samples with high bulk concentrations of polarized spins, the resonance frequencies of individual spins are shifted due to the presence of a non-negligible dipolar field due to spins that are not excited by either the observer or the pump pulses. This field leads to frequency shifts [Bibr bib1.bibx13]. Frequency shifts up to 11 MHz have been observed in a crystal of the organic radical 1,3-bisdiphenylene-2-phenylallyl with EPR at 240 GHz and 8.56 T [Bibr bib1.bibx42]. In our samples, the concentration of these spins (which might be termed C spins) is about 1 mM, so the demagnetization field and any associated effects are likely negligible.

## 5 Conclusions

Low temperatures and high fields lead to substantial thermal spin polarization. We showed theoretically that this leads to a hitherto unappreciated and unobserved additional phase factor in the DEER signal from a background of a uniform three-dimensional distribution of spins, resulting in a non-zero out-of-phase signal whose amplitude is proportional to the spin polarization. Experimental data, over a range of thermal spin polarizations, i.e., at several temperatures and magnetic fields, for both monoradical and biradical samples confirm the theoretical model describing this polarization-dependent phase factor.

The theory quantitatively matches the experiments for the intramolecular DEER signal from a biradical, and the observed amplitude of the out-of-phase component of the intermolecular signal contribution is more intense than predicted by current theory. The origin of this discrepancy is unclear. One possibility is that B–B flip–flop terms are responsible. To test this experimentally, spin concentration must be lowered. However, it is unfeasible with current instruments to collect data at much lower concentrations than those shown here, since this would also necessarily require much longer trace lengths to capture a majority of the signal decay. Beyond the reduced number of spins, the signal-to-noise ratio would be additionally degraded by de-coherence. Extending the model beyond the dilute limit to a fully coupled spin network to examine the impact of B–B spin coupling would be the next step in narrowing down the origin of the discrepancy.

Although derived for the case of two spins- 1/2 , the model is also applicable to high-spin systems as long as only one transition of the pump spin is excited by the pump pulse.

The polarization-phase factor is negligible for DEER experiments performed at 50–80 K and 0.35–1.25 T, temperatures and fields typically used for DEER experiments of nitroxide-labeled proteins, since the spin polarization is at most 1.6 % under these conditions. However, the results are potentially relevant for measurements at lower temperatures and higher fields (such as for Gd spin labels measured at 10 K and the W band) and for situations with strong non-thermal polarization such as photo-induced excited-state triplets and optically pumped ground-state triplets.

## Supplement

10.5194/mr-3-101-2022-supplement The supplement related to this article is available online at: https://doi.org/10.5194/mr-3-101-2022-supplement.

## Data Availability

The EPR data used in the main text and Supplement are available at https://doi.org/10.5281/zenodo.6537282 (Sweger et al., 2022).

## References

[bib1.bibx1] Abdullin D, Brehm P, Fleck N, Spicher S, Grimme S, Schiemann O (2019). Pulsed EPR dipolar spectroscopy on spin pairs with one highly anisotropic spin center: the low-spin FeIII case. Eur J Chem.

[bib1.bibx2] Barth K, Hank S, Spindler PE, Prisner TF, Tampé R, Joseph B (2018). Conformational coupling and trans-inhibition in the human antigen transporter ortholog TmrAB resolved with dipolar EPR spectroscopy. J Am Chem Soc.

[bib1.bibx3] Bode BE, Margraf D, Plackmeyer J, Dürner G, Prisner TF, Schiemann O (2007). Counting the monomers in nanometer-sized oligomers by pulsed electron–electron double resonance. J Am Chem Soc.

[bib1.bibx4] Born A, Soetbeer J, Breitgoff F, Henen MA, Sgourakis N, Polyhach Y, Nichols PJ, Strotz D, Jeschke G, Vögeli B (2021). Reconstruction of coupled intra-and interdomain protein motion from nuclear and electron magnetic resonance. J Am Chem Soc.

[bib1.bibx5] Bowen AM, Jones MW, Lovett JE, Gaule TG, McPherson MJ, Dilworth JR, Timmel CR, Harmer JR (2016). Exploiting orientation-selective DEER: determining molecular structure in systems containing Cu (II) centres. Phys Chem Chem Phys.

[bib1.bibx6] Canarie ER, Jahn SM, Stoll S (2020). Quantitative structure-based prediction of electron spin decoherence in organic radicals. J Phys Chem Lett.

[bib1.bibx7] Dal Farra MG, Ciuti S, Gobbo M, Carbonera D, Di Valentin M (2019). Triplet-state spin labels for highly sensitive pulse dipolar spectroscopy. Mol Phys.

[bib1.bibx8] Dal Farra MG, Martin C, Bergantino E, Kandrashkin YE, van der Est A, Di Valentin M (2020). Electron spin polarization transfer induced by triplet–radical interactions in the weakly coupled regime. Phys Chem Chem Phys.

[bib1.bibx9] Dalaloyan A, Qi M, Ruhtstein S, Vega S, Godt A, Feintuch A, Goldfarb D (2015). Gd(III)–Gd(III) EPR distance measurements – the range of accessible distances and the impact of zero field splitting. Phys Chem Chem Phys.

[bib1.bibx10] Dastvan R, Mishra S, Peskova YB, Nakamoto RK, Mchaourab HS (2019). Mechanism of allosteric modulation of P-glycoprotein by transport substrates and inhibitors. Science.

[bib1.bibx11] Di Valentin M, Dal Farra MG, Galazzo L, Albertini M, Schulte T, Hofmann E, Carbonera D (2016). Distance measurements in peridinin-chlorophyll 
a
-protein by light-induced PELDOR spectroscopy. Analysis of triplet state localization. Biochim Biophys Acta Bioenerg.

[bib1.bibx12] Duss O, Yulikov M, Jeschke G, Allain FHT (2014). EPR-aided approach for solution structure determination of large RNAs or protein–RNA complexes. Nat Commun.

[bib1.bibx13] Edzes HT (1990). The nuclear magnetization as the origin of transient changes in the magnetic field in pulsed NMR experiments. J Magn Reson.

[bib1.bibx14] Evans EGB, Morgan JLW, DiMaio F, Zagotta WN, Stoll S (2020). Allosteric conformational change of a cyclic nucleotide-gated ion channel revealed by DEER spectroscopy. Proc Natl Acad Sci USA.

[bib1.bibx15] Hertel MM, Denysenkov VP, Bennati M, Prisner T (2005). Pulsed 180-GHz EPR/ENDOR/PELDOR spectroscopy. Magn Reson Chem.

[bib1.bibx16] Jeschke G (2012). DEER distance measurements on proteins. Annu Rev Phys Chem.

[bib1.bibx17] Jeschke G, Polyhach Y (2007). Distance measurements on spin-labelled biomacromolecules by pulsed electron paramagnetic resonance. Phys Chem Chem Phys.

[bib1.bibx18] Krumkacheva OA, Timofeev IO, Politanskaya LV, Polienko Y, Tretyakov E, Rogozhnikova OY, Trukhin DV, Tormyshev V, Chubarov A, Bagryanskaya E, Fedin M (2019). Triplet fullerenes as prospective spin labels for nanoscale distance measurements by pulsed dipolar EPR spectroscopy. Angew Chem Int Ed.

[bib1.bibx19] Larsen RG, Singel DJ (1993). Double electron–electron resonance spin–echo modulation: Spectroscopic measurement of electron spin pair separations in orientationally disordered solids. J Chem Phys.

[bib1.bibx20] Manglik A, Kim TH, Masureel M, Altenbach C, Yang Z, Hilger D, Lerch MT, Kobilka TS, Thian FS, Hubbell WL, Prosser R, Kobilka B (2015). Structural insights into the dynamic process of 
$\beta_{2}$
-adrenergic receptor signaling. Cell.

[bib1.bibx21] Manukovsky N, Feintuch A, Kuprov I, Goldfarb D (2017). Time domain simulation of Gd
${}^{3+}$
–Gd
${}^{3+}$
 distance measurements by EPR. J Chem Phys.

[bib1.bibx22] Marion DJ-Y, Huber G, Dubois L, Berthault P, Desvaux H (2007). ${}^{1}$
H and 
${}^{129}$
Xe NMR absorption line shapes in the presence of highly polarized and concentrated xenon solutions in high magnetic field. J Magn Reson.

[bib1.bibx23] Marko A, Denysenkov V, Prisner TF (2013). Out-of-phase PELDOR. Mol Phys.

[bib1.bibx24] Maryasov A, Dzuba S, Salikhov K (1982). Spin-polarization effects on the phase relaxation induced by dipole-dipole interactions. J Magn Reson.

[bib1.bibx25] Milov AD, Tsvetkov YD (1997). Double electron–electron resonance in electron spin echo: conformations of spin-labeled poly-4-vinylpyridine in glassy solutions. Appl Magn Reson.

[bib1.bibx26] Milov AD, Salikhov KM, Tsvetkov YD (1973). Effect of spin dipole–dipole interaction on phase relaxation in magnetically dilute solid bodies. J Exp Theor Phys.

[bib1.bibx27] Milov AD, Salikhov KM, Shchirov MD (1981). Application of the double resonance method to electron spin echo in a study of the spatial distribution of paramagnetic centers in solids. Phys Solid State.

[bib1.bibx28] Rohrer M, Brügmann O, Kinzer B, Prisner T (2001). High-field/high-frequency EPR spectrometer operating in pulsed and continuous-wave mode at 180 GHz. Appl Magn Reson.

[bib1.bibx29] Salikhov KM, Kandrashkin YE, Salikhov AK (1992). Peculiarities of Free Induction and Primary Spin Echo Signals for Spin-Correlated Radical Pairs. Appl Magn Reson.

[bib1.bibx30] Schiemann O, Prisner TF (2007). Long-range distance determinations in biomacromolecules by EPR spectroscopy. Q Rev Biophys.

[bib1.bibx31] Schöps P, Spindler P, Marko A, Prisner T (2015). Broadband spin echoes and broadband SIFTER in EPR. J Magn Reson.

[bib1.bibx32] Schöps P, Plackmeyer J, Marko A (2016). Separation of intra-and intermolecular contributions to the PELDOR signal. J Magn Reson.

[bib1.bibx33] Shi F, Zhang Q, Wang P, Sun H, Wang J, Rong X, Chen M, Ju C, Reinhard F, Chen H, Wrachtrup J, Wang J, Du J (2015). Single-protein spin resonance spectroscopy under ambient conditions. Science.

[bib1.bibx34] Stepanov V, Takahashi S (2016). Determination of nitrogen spin concentration in diamond using double electron-electron resonance. Phys Rev B.

[bib1.bibx35] Sushkov AO, Lovchinsky I, Chisholm N, Walsworth RL, Park H, Lukin MD (2014). Magnetic resonance detection of individual proton spins using quantum reporters. Phys Rev Lett.

[bib1.bibx36] Sweger SR, Denysenkov VP, Maibaum L, Prisner TF, Stoll S (2022). Zenodo.

[bib1.bibx37] Tait CE, Stoll S (2016). Coherent pump pulses in double electron electron resonance spectroscopy. Phys Chem Chem Phys.

[bib1.bibx38] Takahashi S, Hanson R, Van Tol J, Sherwin MS, Awschalom DD (2008). Quenching spin decoherence in diamond through spin bath polarization. Phys Rev Lett.

[bib1.bibx39] Tang J, Thurnauer MC, Norris JR (1994). Electron spin echo envelope modulation due to exchange and dipolar interactions in a spin-correlated radical pair. Chem Phys Lett.

[bib1.bibx40] Tavenor NA, Silva KI, Saxena S, Horne SW (2014). Origins of structural flexibility in protein-based supramolecular polymers revealed by DEER spectroscopy. J Phys Chem B.

[bib1.bibx41] Verhalen B, Dastvan R, Thangapandian S, Peskova Y, Koteiche HA, Nakamoto RK, Tajkhorshid E, Mchaourab HS (2017). Energy transduction and alternating access of the mammalian ABC transporter P-glycoprotein. Nature.

[bib1.bibx42] Wilson CB, Edwards DT, Clayton JA, Han S, Sherwin MS (2020). Dressed Rabi oscillation in a crystalline organic radical. Phys Rev Lett.

