# Supplementary material for: The effect of spin polarization on double electron–electron resonance (DEER) spectroscopy
Source: Magn Reson (Gott). 2022 Jul 1;3(1):101–10. doi: 10.5194/mr-3-101-2022 (PMC10583274; doi:10.5194/mr-3-101-2022)
Supplement: The supplement related to this article is available online at: https://doi.org/10.5194/mr-3-101-2022-supplement. [file mr-3-101-supplement.pdf]

Supplement of Magn. Reson., 3, 101–110, 2022
https://doi.org/10.5194/mr-3-101-2022-supplement
© Author(s) 2022. CC BY 4.0 License.

*Supplement of*

# The effect of spin polarization on double electron–electron resonance (DEER) spectroscopy

**Sarah R. Sweger et al.**

*Correspondence to:* Stefan Stoll (stst@uw.edu)

The copyright of individual parts of the supplement might differ from the article licence.

# Contents

**S1 Theoretical DEER signal from a single A–B spin pair** **1**

**S2 Theoretical DEER signal from A-spins in a homogeneous distribution of B-spins** **4**
S2.1 The cos integral . . . . . 5
S2.2 The sin integral . . . . . 6

**S3 Numerical calculations** **7**
S3.1 Monte Carlo simulation of intermolecular signal . . . . . 7
S3.2 Nearest-neighbor distance distribution . . . . . 7

**S4 Fit parameters** **9**

**S5 Experimental validation** **9**

# S1 Theoretical DEER signal from a single A–B spin pair

Here we derive the 3-pulse DEER signal from a single A–B spin pair, without making the high-temperature approximation. The recapitulates the derivation by Marko et al (Marko et al., 2013), with adjusted notation. Assuming that the effect of the hyperfine interactions can be folded into the resonance frequencies of the individual spins, the spin Hamiltonian contains terms for the Zeeman and dipolar interactions

$$\hat{H}_{\mathrm{lab}} = \hat{H}_{\mathrm{Z}} + \hat{H}_{\mathrm{D}} \tag{S1}$$

In the high-field limit, the Zeeman interaction term expands to

$$\hat{H}_{\mathrm{Z}} = \hbar\omega_{\mathrm{A}}\hat{S}_{\mathrm{Az}} + \hbar\omega_{\mathrm{B}}\hat{S}_{\mathrm{Bz}} \tag{S2}$$

where $\hbar$ is the reduced Planck constant, $\omega_{\mathrm{A}}$ and $\omega_{\mathrm{B}}$ are the resonance angular frequencies of the two spins, and $\hat{S}_{Az}$ and $\hat{S}_{Bz}$ are the spin operators. In the high-field limit, the dipolar interaction term is

$$\hat{H}_{\mathrm{D}} = \hbar\omega_{\mathrm{AB}}\hat{S}_{\mathrm{Az}}\hat{S}_{\mathrm{Bz}} \tag{S3}$$

where $\omega_{\mathrm{AB}}$ is the dipolar coupling angular frequency

$$\omega_{\mathrm{AB}} = D\,\frac{1-3\cos^2\theta_{\mathrm{AB}}}{r_{\mathrm{AB}}^3} \tag{S4}$$

with the dipolar constant

$$D = \frac{\mu_0}{4\pi}\frac{\mu_{\mathrm{B}}^2 g_{\mathrm{e}}^2}{\hbar} \approx 2\pi\cdot 52.04\,\mathrm{MHz\,nm^3} \tag{S5}$$

where $\mu_0$ is the magnetic constant, $\mu_{\mathrm{B}}$ is the Bohr magneton, $g_{\mathrm{e}}$ is g-value of the free electron, $r_{\mathrm{AB}}$ is the length of the interspin vector, and $\theta_{\mathrm{AB}}$ is the orientation of interspin vector with respect to the external magnetic field, $B_0$.

In order to calculate the DEER signal, it is convenient to transform the Hamiltonian from the laboratory frame to a rotating frame that is rotating with angular frequency $\omega$ around the $z$-axis, where $\omega$ is the detection angular frequency

$$\hat{H} = \exp(\mathrm{i}\omega t(\hat{S}_{\mathrm{Az}}+\hat{S}_{\mathrm{Bz}}))\hat{H}_{\mathrm{lab}}\exp(-\mathrm{i}\omega t(\hat{S}_{\mathrm{Az}}+\hat{S}_{\mathrm{Bz}})) - \hbar\omega(\hat{S}_{\mathrm{Az}}+\hat{S}_{\mathrm{Bz}}) \tag{S6}$$

This gives

$$\hat{H} = \hbar(\Delta\omega_{\mathrm{A}}\hat{S}_{\mathrm{Az}} + \Delta\omega_{\mathrm{B}}\hat{S}_{\mathrm{Bz}} + \omega_{\mathrm{AB}}\hat{S}_{\mathrm{Az}}\hat{S}_{\mathrm{Bz}}) \tag{S7}$$

where $\Delta\omega_i = \omega_i - \omega$ are the angular frequency offsets of the two spins ($i = \mathrm{A, B}$).

Deriving the DEER signal requires propagating the density operator through the course of the DEER experiment, beginning from the equilibrium density

$$\hat{\sigma}_{\mathrm{eq}} = \frac{\exp(-\hat{H}_{\mathrm{Z}}/k_{\mathrm{B}}T)}{Z} \tag{S8}$$

where $k_\mathrm{B}$ is the Boltzmann constant, $T$ is the temperature, and $Z = \mathrm{tr}(\exp(-\hat{H}_\mathrm{Z}/k_\mathrm{B}T))$ is the partition function. In Eq. (S8), we have neglected the dipolar coupling term $\hat{H}_\mathrm{D}$ in the Hamiltonian, which is much smaller than the two Zeeman terms. Next, we use

$$\exp(-\hbar\omega_i \hat{S}_{iz}/k_\mathrm{B}T) = \hat{\mathbb{1}}\cosh\left(\frac{\hbar\omega_i}{2k_\mathrm{B}T}\right) - 2\hat{S}_{iz}\sinh\left(\frac{\hbar\omega_i}{2k_\mathrm{B}T}\right) = \hat{\mathbb{1}}c_i - 2\hat{S}_{iz}s_i \tag{S9}$$

where $\hat{\mathbb{1}}$ is the identity operator, and $c_i$ and $s_i$ are abbreviations for the cosh and sinh factors. With this, we get

$$\begin{aligned}\hat{\sigma}_\mathrm{eq} &= \frac{\hat{\mathbb{1}}c_\mathrm{A}c_\mathrm{B} - 2\hat{S}_\mathrm{Bz}c_\mathrm{A}s_\mathrm{B} - 2\hat{S}_\mathrm{Az}c_\mathrm{B}s_\mathrm{A} + 4\hat{S}_\mathrm{Az}\hat{S}_\mathrm{Bz}s_\mathrm{A}s_\mathrm{B}}{4c_\mathrm{A}c_\mathrm{B}} \\ &= \frac{1}{4}\hat{\mathbb{1}} - \frac{1}{2}\epsilon_\mathrm{A}\hat{S}_{\mathrm{A}z} - \frac{1}{2}\epsilon_\mathrm{B}\hat{S}_{\mathrm{B}z} + \frac{1}{2}\cdot\epsilon_\mathrm{A}\epsilon_\mathrm{B}\cdot 2\hat{S}_{\mathrm{A}z}\hat{S}_{\mathrm{B}z}\end{aligned} \tag{S10}$$

where

$$\epsilon_i = \tanh\left(\frac{\hbar\omega_i}{2k_\mathrm{B}T}\right) \tag{S11}$$

is the thermal spin polarization of spin $i$.

The evolution in time of the density matrix is governed by the Liouville–von Neumann equation. In its integrated form, it is

$$\hat{\sigma}(t) = \hat{U}(t)\hat{\sigma}_\mathrm{eq}\hat{U}^\dagger(t) \tag{S12}$$

where $\hat{U}$ is the total propagator of the DEER sequence and $t$ is the pump pulse position. In the rotating frame and with ideal pulses, the Hamiltonian is piece-wise constant, and therefore the propagator is a composition of simple exponential operators:

$$\hat{U}(t) = \mathrm{e}^{-\mathrm{i}\hat{H}\tau}\mathrm{e}^{-\mathrm{i}\pi\hat{S}_{\mathrm{A}y}}\mathrm{e}^{-\mathrm{i}\hat{H}(\tau-t)}\mathrm{e}^{-\mathrm{i}\pi\hat{S}_{\mathrm{B}y}}\mathrm{e}^{-\mathrm{i}\hat{H}t}\mathrm{e}^{-\mathrm{i}(\pi/2)\hat{S}_{\mathrm{A}y}} \tag{S13}$$

For describing the pulses, we assume ideal pulses that are selective for A or B spins, and we neglect fast oscillating terms and internal interactions during the pulses. All pulses in $\hat{U}(t)$ are set to have $y$ phase. A more visual way to represent the propagator is

$$U(t) = \xrightarrow{(\pi/2)\hat{S}_{\mathrm{A}y}}\xrightarrow{\hat{H}t}\xrightarrow{\pi\hat{S}_{\mathrm{B}y}}\xrightarrow{\hat{H}(\tau-t)}\xrightarrow{\pi\hat{S}_{\mathrm{A}y}}\xrightarrow{\hat{H}\tau} \tag{S14}$$

Before applying the propagator to the starting density based on Eq. (S12), we simplify the propagator. This is possible due the simplicity of the rotating-frame Hamiltonian $\hat{H}$, which only contains terms with $\hat{S}_{\mathrm{A}z}$ and $\hat{S}_{\mathrm{B}z}$ that all mutually commute.

First, inserting the identity operator $\mathbb{1} = \mathrm{e}^{+\mathrm{i}\pi\hat{S}_{\mathrm{B}y}}\mathrm{e}^{-\mathrm{i}\pi\hat{S}_{\mathrm{B}y}}$ to the right of the $\hat{H}t$ propagator gives

$$U(t) = \mathrm{e}^{-\mathrm{i}\hat{H}\tau}\mathrm{e}^{-\mathrm{i}\pi\hat{S}_{\mathrm{A}y}}\mathrm{e}^{-\mathrm{i}\hat{H}(\tau-t)}\underbrace{\mathrm{e}^{-\mathrm{i}\pi\hat{S}_{\mathrm{B}y}}\mathrm{e}^{-\mathrm{i}\hat{H}t}\mathrm{e}^{+\mathrm{i}\pi\hat{S}_{\mathrm{B}y}}}\mathrm{e}^{-\mathrm{i}\pi\hat{S}_{\mathrm{B}y}}\mathrm{e}^{-\mathrm{i}(\pi/2)\hat{S}_{\mathrm{A}x}} \tag{S15}$$

Next, the bracketed propagator combination can be written as $\mathrm{e}^{-\mathrm{i}\hat{H}_\mathrm{B}t}$, where $\hat{H}_\mathrm{B}$ is the transformed Hamiltonian

$$\hat{H}_\mathrm{B} = \mathrm{e}^{-\mathrm{i}\pi\hat{S}_{\mathrm{B}y}}\hat{H}\mathrm{e}^{+\mathrm{i}\pi\hat{S}_{\mathrm{B}y}} = \Delta\omega_\mathrm{A}\hat{S}_{\mathrm{A}z} - \Delta\omega_\mathrm{B}\hat{S}_{\mathrm{B}z} - \omega_\mathrm{AB}\hat{S}_\mathrm{Az}\hat{S}_\mathrm{Bz} \tag{S16}$$

This is possible since, in general, $\hat{U}\mathrm{e}^{-\mathrm{i}\hat{A}}\hat{U}^\dagger = \mathrm{e}^{-\mathrm{i}\hat{U}\hat{A}\hat{U}^\dagger}$.

Since $\hat{H}$ and $\hat{H}_\mathrm{B}$ commute (as both contain only $z$ spin operators), we combine them into a single propagator exponent. The total propagator now reads

$$\hat{U}(t) = \mathrm{e}^{-\mathrm{i}\hat{H}\tau}\mathrm{e}^{-\mathrm{i}\pi\hat{S}_{\mathrm{A}y}}\mathrm{e}^{-\mathrm{i}(\hat{H}(\tau-t)+\hat{H}_\mathrm{B}t)}\mathrm{e}^{-\mathrm{i}\pi\hat{S}_{\mathrm{B}y}}\mathrm{e}^{-\mathrm{i}(\pi/2)\hat{S}_{\mathrm{A}y}} \tag{S17}$$

Inserting the identity operator $\mathbb{1} = \mathrm{e}^{+\mathrm{i}\pi\hat{S}_{\mathrm{A}y}}\mathrm{e}^{-\mathrm{i}\pi\hat{S}_{\mathrm{A}y}}$ to the right of the $\hat{H}/\hat{H}_\mathrm{B}$ propagator yields

$$\hat{U}(t) = \mathrm{e}^{-\mathrm{i}\hat{H}\tau}\underbrace{\mathrm{e}^{-\mathrm{i}\pi\hat{S}_{\mathrm{A}y}}\mathrm{e}^{-\mathrm{i}(\hat{H}(\tau-t)+\hat{H}_\mathrm{B}t}\mathrm{e}^{+\mathrm{i}\pi\hat{S}_{\mathrm{A}y}}}\underbrace{\mathrm{e}^{-\mathrm{i}\pi\hat{S}_{\mathrm{A}y}}\mathrm{e}^{-\mathrm{i}\pi\hat{S}_{\mathrm{B}y}}\mathrm{e}^{-\mathrm{i}(\pi/2)\hat{S}_{\mathrm{A}y}}} \tag{S18}$$

The propagator combination of the right bracket simplifies to $\mathrm{e}^{-\mathrm{i}\pi\hat{S}_{\mathrm{B}y}}\mathrm{e}^{-\mathrm{i}(3\pi/2)\hat{S}_{\mathrm{A}y}}$, since $\hat{S}_{\mathrm{A}y}$ and $\hat{S}_{\mathrm{B}y}$ commute. The propagator combination of the left bracket can again be rewritten with the transformed Hamiltonians:

$$\hat{U}(t) = \mathrm{e}^{-\mathrm{i}\hat{H}\tau}\mathrm{e}^{-\mathrm{i}(\hat{H}_{\mathrm{A}}(\tau-t)+\hat{H}_{\mathrm{AB}}t}\mathrm{e}^{-\mathrm{i}\pi\hat{S}_{\mathrm{B}y}}\mathrm{e}^{-\mathrm{i}(3\pi/2)\hat{S}_{\mathrm{A}y}} \tag{S19}$$

where

$$\hat{H}_{\mathrm{A}} = \mathrm{e}^{-\mathrm{i}\pi\hat{S}_{\mathrm{A}y}}\hat{H}\mathrm{e}^{+\mathrm{i}\pi\hat{S}_{\mathrm{A}y}} = -\Delta\omega_{\mathrm{A}}\hat{S}_{\mathrm{A}z} + \Delta\omega_{\mathrm{B}}\hat{S}_{\mathrm{B}z} - \omega_{\mathrm{AB}}\hat{S}_{\mathrm{A}z}\hat{S}_{\mathrm{B}z} \tag{S20}$$

$$\hat{H}_{\mathrm{AB}} = \mathrm{e}^{-\mathrm{i}\pi\hat{S}_{\mathrm{A}y}}\hat{H}_{\mathrm{B}}\mathrm{e}^{+\mathrm{i}\pi\hat{S}_{\mathrm{A}y}} = -\Delta\omega_{\mathrm{A}}\hat{S}_{\mathrm{A}z} - \Delta\omega_{\mathrm{B}}\hat{S}_{\mathrm{B}z} + \omega_{\mathrm{AB}}\hat{S}_{\mathrm{A}z}\hat{S}_{\mathrm{B}z} \tag{S21}$$

After combining the adjacent exponential operators with the Hamiltonians in the exponent, we get

$$\hat{U}(t) = \mathrm{e}^{-\mathrm{i}(\hat{H}\tau+\hat{H}_{\mathrm{A}}(\tau-t)+\hat{H}_{\mathrm{AB}}t)}\mathrm{e}^{-\mathrm{i}\pi\hat{S}_{\mathrm{B}y}}\mathrm{e}^{-\mathrm{i}(3\pi/2)\hat{S}_{\mathrm{A}y}} \tag{S22}$$

The sum of the Hamiltonian terms is

$$\hat{H}\tau + \hat{H}_{\mathrm{A}}(\tau-t) + \hat{H}_{\mathrm{AB}}t = 2\Delta\omega_{\mathrm{B}}(\tau-t)\hat{S}_{\mathrm{B}z} + \omega_{\mathrm{AB}}t\cdot 2\hat{S}_{\mathrm{A}z}\hat{S}_{\mathrm{B}z} \tag{S23}$$

Since $\hat{S}_{\mathrm{B}z}$ and $2\hat{S}_{\mathrm{A}z}\hat{S}_{\mathrm{B}z}$ commute, we can separate the propagator into two, one for each term. With this, the final expression for the total propagator is

$$\hat{U}(t) = \mathrm{e}^{-\mathrm{i}\omega_{\mathrm{AB}}t\,2\hat{S}_{\mathrm{A}z}\hat{S}_{\mathrm{B}z}}\mathrm{e}^{-\mathrm{i}2\Delta\omega_{\mathrm{B}}(\tau-t)\hat{S}_{\mathrm{B}z}}\mathrm{e}^{-\mathrm{i}\pi\hat{S}_{\mathrm{B}y}}\mathrm{e}^{-\mathrm{i}(3\pi/2)\hat{S}_{\mathrm{A}y}} \tag{S24}$$

or, more visually,

$$\hat{U}(t) = \xrightarrow{(3\pi/2)\hat{S}_{\mathrm{A}y}}\xrightarrow{\pi\hat{S}_{\mathrm{B}y}}\xrightarrow{2\Delta\omega_{\mathrm{B}}(\tau-t)\hat{S}_{\mathrm{B}z}}\xrightarrow{\omega_{\mathrm{AB}}t\,2\hat{S}_{\mathrm{A}z}\hat{S}_{\mathrm{B}z}} \tag{S25}$$

Next, to get the final density $\sigma(t)$, we apply this propagator to the starting density from Eq. (S10):

$$\begin{aligned}
\hat{\sigma}_{\mathrm{eq}} =& \frac{\hat{\mathbb{1}}}{4} - \frac{\epsilon_{\mathrm{A}}}{2}\hat{S}_{\mathrm{A}z} - \frac{\epsilon_{\mathrm{B}}}{2}\hat{S}_{\mathrm{B}z} + \frac{\epsilon_{\mathrm{A}}\epsilon_{\mathrm{B}}}{2}\cdot 2\hat{S}_{\mathrm{A}z}\hat{S}_{\mathrm{B}z} \xrightarrow{(3\pi/2)\hat{S}_{\mathrm{A}y}} \\
& \frac{\hat{\mathbb{1}}}{4} + \frac{\epsilon_{\mathrm{A}}}{2}\hat{S}_{\mathrm{A}x} - \frac{\epsilon_{\mathrm{B}}}{2}\hat{S}_{\mathrm{B}z} - \frac{\epsilon_{\mathrm{A}}\epsilon_{\mathrm{B}}}{2}\cdot 2\hat{S}_{\mathrm{A}x}\hat{S}_{\mathrm{B}z} \xrightarrow{\pi\hat{S}_{\mathrm{B}y}} \\
& \frac{\hat{\mathbb{1}}}{4} + \frac{\epsilon_{\mathrm{A}}}{2}\hat{S}_{\mathrm{A}x} + \frac{\epsilon_{\mathrm{B}}}{2}\hat{S}_{\mathrm{B}z} + \frac{\epsilon_{\mathrm{A}}\epsilon_{\mathrm{B}}}{2}\cdot 2\hat{S}_{\mathrm{A}x}\hat{S}_{\mathrm{B}z} \xrightarrow{2\Delta\omega_{\mathrm{B}}(\tau-t)\hat{S}_{\mathrm{B}z}} \\
& \frac{\hat{\mathbb{1}}}{4} + \frac{\epsilon_{\mathrm{A}}}{2}\hat{S}_{\mathrm{A}x} + \frac{\epsilon_{\mathrm{B}}}{2}\hat{S}_{\mathrm{B}z} + \frac{\epsilon_{\mathrm{A}}\epsilon_{\mathrm{B}}}{2}\cdot 2\hat{S}_{\mathrm{A}x}\hat{S}_{\mathrm{B}z} \xrightarrow{\omega_{\mathrm{AB}}t\,2\hat{S}_{\mathrm{A}z}\hat{S}_{\mathrm{B}z}} \\
& \frac{\hat{\mathbb{1}}}{4} + \frac{\epsilon_{\mathrm{A}}}{2}\hat{S}_{\mathrm{A}x}\cos(\omega_{\mathrm{AB}}t) + \frac{\epsilon_{\mathrm{A}}}{2}\cdot 2\hat{S}_{\mathrm{A}y}\hat{S}_{\mathrm{B}z}\sin(\omega_{\mathrm{AB}}t) + \frac{\epsilon_{\mathrm{B}}}{2}\hat{S}_{\mathrm{B}z} \\
& \quad + \frac{\epsilon_{\mathrm{A}}\epsilon_{\mathrm{B}}}{2}\cdot 2\hat{S}_{\mathrm{A}x}\hat{S}_{\mathrm{B}z}\cos(\omega_{\mathrm{AB}}t) + \frac{\epsilon_{\mathrm{A}}\epsilon_{\mathrm{B}}}{2}\cdot\hat{S}_{\mathrm{A}y}\sin(\omega_{\mathrm{AB}}t) = \hat{\sigma}(t)
\end{aligned} \tag{S26}$$

(Note that the $\hat{S}_{\mathrm{B}z}$ propagator has left the density unchanged.)

The echo signal is

$$V(t) = \mathrm{tr}(\hat{S}_{\mathrm{A}+}\hat{\sigma}(t)) \tag{S27}$$

The only two terms from the final density in Eq. (S26) that give a non-zero trace with $\hat{S}_{\mathrm{A}+}$ are those containing $\hat{S}_{\mathrm{A}x}$ and $\hat{S}_{\mathrm{A}y}$, since $\mathrm{tr}(\hat{S}_{\mathrm{A}+}\hat{S}_{\mathrm{A}x}) = 1$ and $\mathrm{tr}(\hat{S}_{\mathrm{A}+}\hat{S}_{\mathrm{A}y}) = \mathrm{i}$. Therefore, only these terms survive:

$$V(t) = \frac{\epsilon_{\mathrm{A}}}{2}\cos(\omega_{\mathrm{AB}}t) + \mathrm{i}\frac{\epsilon_{\mathrm{A}}\epsilon_{\mathrm{B}}}{2}\sin(\omega_{\mathrm{AB}}t) = \frac{\epsilon_{\mathrm{A}}}{2}\left[\cos(\omega_{\mathrm{AB}}t) + \mathrm{i}\epsilon_{\mathrm{B}}\sin(\omega_{\mathrm{AB}}t)\right] \tag{S28}$$

Dropping the prefactor (which is absorbed into the overall amplitude factor $V_0$), we get the final result

$$V(t) = \cos(\omega_{\mathrm{AB}}t) + \mathrm{i}\epsilon_{\mathrm{B}}\sin(\omega_{\mathrm{AB}}t) \tag{S29}$$

In a thermally equilibrated sample, the polarization of pump and probe spins are identical, therefore we can set $\epsilon = \epsilon_{\mathrm{a}} = \epsilon_{\mathrm{B}}$.

## S2 Theoretical DEER signal from A-spins in a homogeneous distribution of B-spins

For a sample with an A-spin in a uniform, random, spatial distribution of B-spins, the total signal is a product of all individual $V_{\mathrm{AB}}$ signals from Eq. (S29), additionally averaged over all B-spin configurations

$$V_{\mathrm{inter}}(t) = \left\langle \prod_{b=1}^{N_{\mathrm{B}}} V_{\mathrm{AB}}(\boldsymbol{r}_b, t) \right\rangle \tag{S30}$$

Here, $N_{\mathrm{B}}$ is the number of B-spins in a configuration, $\boldsymbol{r}_b$ is the vector from the A-spin to the $b$th B-spin, and the angled brackets indicate an average over B-spin configurations. To arrive at this product form, the dipolar couplings among B spins are neglected.

With the assumption that the positions of the B-spins are uncorrelated, the averaged product can be replaced with the product of the averages.

$$V_{\mathrm{inter}}(t) = \prod_{b=1}^{N_{\mathrm{B}}} \langle V_{\mathrm{AB}}(\boldsymbol{r}_b, t) \rangle \tag{S31}$$

Additionally, with the assumption that all B-spins are equally distributed, all averages are equal and independent of $b$, yielding

$$V_{\mathrm{inter}}(t) = \langle V_{\mathrm{AB}}(\boldsymbol{r}, t) \rangle^{N_{\mathrm{B}}} \tag{S32}$$

Defining a (large) spherical region $S_R$ with a radius $R$, $V_{\mathrm{inter}}(t)$ is obtained by calculating the signal for all $N_{\mathrm{B}}$ B-spins within $S_R$ and then taking the limit $R \to \infty$

$$V_{\mathrm{inter}}(t) = \lim_{R\to\infty} \left(\langle V_{\mathrm{AB}}(\boldsymbol{r}, t) \rangle_R\right)^{N_{\mathrm{B}}} \tag{S33}$$

with

$$\langle V_{\mathrm{AB}}(\boldsymbol{r}, t) \rangle_R = 1 + p_{\mathrm{B}} \langle \cos(\omega t) - 1 \rangle_R + \mathrm{i}\epsilon p_{\mathrm{B}} \langle \sin(\omega t) \rangle_R \tag{S34}$$

based on Eq. (S29). The averages over the oscillatory terms are

$$\langle \cos(\omega t) - 1 \rangle_R = \frac{1}{V_R} \int_{S_R} \mathrm{d}\boldsymbol{r} \left( \cos(\omega(\boldsymbol{r})t) - 1 \right) \equiv \frac{1}{V_R} C_R(t) \tag{S35}$$

$$\langle \sin(\omega t) \rangle_R = \frac{1}{V_R} \int_{S_R} \mathrm{d}\boldsymbol{r} \, \sin(\omega(\boldsymbol{r})t) \equiv \frac{1}{V_R} S_R(t) \tag{S36}$$

where $V_R = \frac{4\pi}{3} R^3$ is the volume of the sphere. The integrals $C_R(t)$ and $S_R(t)$ cannot be evaluated analytically, but if we take the limit $R \to \infty$, the integrals give

$$C(t) = \lim_{R\to\infty} C_R(t) = -\frac{8\pi^2}{9\sqrt{3}} \cdot D|t| \tag{S37}$$

$$S(t) = \lim_{R\to\infty} S_R(t) = \frac{8\pi}{27} \left[ 3 + \sqrt{3} \ln(2 - \sqrt{3}) \right] \cdot Dt \tag{S38}$$

The derivations are given separately below in sections S2.1 and S2.2 below. Note that $C(t)$ is proportional to $|t|$, whereas $S(t)$ is proportional to $t$. Using $C(t)$ and $S(t)$ instead of $C_R(t)$ and $S_R(t)$, Eq. (S34) becomes

$$\langle V_{\mathrm{AB}}(\boldsymbol{r}, t) \rangle_R = 1 + p_{\mathrm{B}} \frac{C(t) + \mathrm{i}\epsilon S(t)}{V_R} \tag{S39}$$

with a small error for finite $R$ that will vanish once we take the limit $R \to \infty$. Given a volume concentration of B-spins of $c_{\mathrm{B}}$, the number of B-spins within $S_R$ is $N_{\mathrm{B}} = c_{\mathrm{B}} V_R$, and the volume is $V_R = N_{\mathrm{B}}/c_{\mathrm{B}}$. Combining this with Eq. (S39) and inserting into Eq. (S33) gives

$$V_{\mathrm{inter}}(t) = \lim_{R\to\infty} \left( 1 + p_{\mathrm{B}} c_{\mathrm{B}} \frac{C(t) + \mathrm{i}\epsilon S(t)}{N_{\mathrm{B}}} \right)^{N_{\mathrm{B}}} \tag{S40}$$

The limit $R \to \infty$ corresponds to $V_R \to \infty$ and $N_\mathrm{B} \to \infty$, so that we get

$$V_\mathrm{inter}(t) = \lim_{N_\mathrm{B}\to\infty} \left(1 + p_\mathrm{B} c_\mathrm{B} \frac{C(t) + \mathrm{i}\epsilon S(t)}{N_\mathrm{B}}\right)^{N_\mathrm{B}} = \exp\left[p_\mathrm{B} c_\mathrm{B} (C(t) + \mathrm{i}\epsilon S(t))\right] \tag{S41}$$

Inserting the expressions for $C(t)$ and $S(t)$ from Eqs. (S37) and (S38) yields the final expression

$$V_\mathrm{inter}(t) = \exp\left(-k|t| + \mathrm{i}\alpha\epsilon k t\right) = \exp(-k|t|) \cdot \exp(\mathrm{i}\alpha\epsilon k t) \tag{S42}$$

where

$$k = \frac{8\pi^2}{9\sqrt{3}} p_\mathrm{B} c_\mathrm{B} D \approx 5.0651\, p_\mathrm{B} c_\mathrm{B} D \qquad \alpha = \frac{\sqrt{3} + \ln(2-\sqrt{3})}{\pi} \approx 0.13213 \tag{S43}$$

## S2.1 The cos integral

Here we solve the $C(t)$ integral from Eq. (S37). Its explicit form is

$$C(t) = \int_0^{2\pi} \mathrm{d}\phi \int_0^{\pi} \mathrm{d}\theta \sin\theta \int_0^{\infty} \mathrm{d}r\, r^2 \left[\cos\left(\frac{Dt}{r^3}(1 - 3\cos^2\theta)\right) - 1\right] \tag{S44}$$

Since the integrand is independent of $\phi$, the $\phi$ integral reduces to a prefactor of $2\pi$. Next, we simplify the notation using the substitution $z = \cos\theta$

$$\int_0^{\pi} \mathrm{d}\theta\, \sin\theta = \int_{-1}^{1} \mathrm{d}\cos\theta = \int_{-1}^{1} \mathrm{d}z \tag{S45}$$

Since the integrand is symmetric about $z = 0$, we can reduce the $z$ integration interval to $[0, 1]$ and prepend a factor of 2. The integral now is

$$C(t) = 4\pi \int_0^1 \mathrm{d}z \int_0^{\infty} \mathrm{d}r\, r^2 \left[\cos\left(\frac{Dt}{r^3}(1 - 3z^2)\right) - 1\right] \tag{S46}$$

Next, we make the substitution $x = r^{-3}$ with

$$\frac{\mathrm{d}x}{\mathrm{d}r} = -\frac{3}{r^4} \qquad \mathrm{d}r = -\frac{r^4}{3}\mathrm{d}x \qquad r^2\mathrm{d}r = -\frac{r^6}{3}\mathrm{d}x = -\frac{1}{3x^2}\mathrm{d}x \qquad \int_0^{\infty} r^2\mathrm{d}r = \frac{1}{3}\int_0^{\infty} x^{-2}\mathrm{d}x \tag{S47}$$

Inserting this substitution gives

$$C(t) = \frac{4\pi}{3} \int_0^1 \mathrm{d}z \int_0^{\infty} \mathrm{d}x\, \frac{\cos(Dt(1 - 3z^2)x) - 1}{x^2} \tag{S48}$$

The integral of $(\cos(ax) - 1)/x^2$ over $x$ can be solved using integration by parts with $u(x) = \cos(ax) - 1$ and $v'(x) = 1/x^2$. This gives

$$\int \mathrm{d}x\, \frac{\cos(ax) - 1}{x^2} = \frac{1 - \cos(ax)}{x} - a \int \mathrm{d}x\, \frac{\sin(ax)}{x} \tag{S49}$$

The first term is zero for both $x = 0$ and $x = \infty$, so that we get

$$\int_0^{\infty} \mathrm{d}x\, \frac{\cos(ax) - 1}{x^2} = -a \int_0^{\infty} \mathrm{d}x\, \frac{\sin(ax)}{x} = -a \cdot \frac{\pi}{2}\mathrm{sgn}(a) = -\frac{\pi}{2}|a| \tag{S50}$$

This gives

$$C(t) = -\frac{2\pi^2}{3}|Dt| \int_0^1 \mathrm{d}z\, |1 - 3z^2| \tag{S51}$$

Evaluating the remaining $z$ integral gives

$$\int_0^1 \mathrm{d}z\, |1 - 3z^2| = \int_0^{1/\sqrt{3}} \mathrm{d}z\, (1 - 3z^2) + \int_{1/\sqrt{3}}^{1} \mathrm{d}z\, (3z^2 - 1) = \frac{2}{3\sqrt{3}} + \frac{2}{3\sqrt{3}} = \frac{4}{3\sqrt{3}} \tag{S52}$$

so that we finally get

$$C(t) = -\frac{2\pi^2}{3}|Dt| \cdot \frac{4}{3\sqrt{3}} = -\frac{8\pi^2}{9\sqrt{3}} \cdot D|t| \tag{S53}$$

where we have also used the fact that $D$ is positive.

## S2.2 The sin integral

Next, we solve the integral $S(t)$ from Eq. (S38). This turned out to be somewhat involved. The explicit form is

$$S(t) = \int_0^{2\pi} \mathrm{d}\phi \int_0^{\pi} \mathrm{d}\theta \sin\theta \int_0^{\infty} \mathrm{d}r\, r^2 \sin\left(\frac{Dt}{r^3}(1 - 3\cos^2\theta)\right) \tag{S54}$$

Again, the $\phi$ integral adds a prefactor of $2\pi$. Applying the same $\cos\theta \to z$ and $1/r^3 \to x$ substitutions as for $C(t)$ gives

$$S(t) = \frac{4\pi}{3} \int_0^1 \mathrm{d}z \int_0^{\infty} \mathrm{d}x\, \frac{\sin\left(Dt(1-3z^2)x\right)}{x^2} \tag{S55}$$

As a result of the $r \to x$ substitution, the integrand now diverges for $x \to 0$. Therefore, we need to be cautious and write the overall integral in terms of a limit:

$$S(t) = \frac{4\pi}{3} \lim_{u\to 0} \int_0^1 \mathrm{d}z \int_u^{\infty} \mathrm{d}x\, \frac{\sin\left(Dt(1-3z^2)x\right)}{x^2} \tag{S56}$$

(In principle, we could already write the initial integral expression with a limit $r_{\mathrm{max}} \to \infty$.) Depending on $t$ and $z$, the argument of the sin function can be positive or negative. To solve the $x$ integral, we need a non-negative argument. Utilizing $\sin(\xi) = \mathrm{sgn}(\xi)\sin|\xi|$, we get

$$S(t) = \frac{4\pi}{3}\mathrm{sgn}(Dt) \lim_{u\to 0} \int_0^1 \mathrm{d}z\, \mathrm{sgn}(1-3z^2) \int_u^{\infty} \mathrm{d}x\, \frac{\sin\left(|Dt||1-3z^2|x\right)}{x^2} \tag{S57}$$

The integral of $\sin(ax)/x^2$ over $x$ can be solved using integration by parts with $u(x) = \sin(ax)$ and $v'(x) = 1/x^2$. For $a \geq 0$, this gives

$$\int \mathrm{d}x\, \frac{\sin(ax)}{x^2} = a\,\mathrm{Ci}(ax) - \frac{\sin(ax)}{x} \tag{S58}$$

with the cosine integral function $\mathrm{Ci}(\cdot)$, defined only for non-negative arguments. For $x \to \infty$, both terms evaluate to 0, so that the overall integral can now be written as

$$S(t) = \frac{4\pi}{3}\mathrm{sgn}(Dt) \lim_{u\to 0} \int_0^1 \mathrm{d}z\, \mathrm{sgn}(1-3z^2) \left[\frac{\sin(|Dt||1-3z^2|u)}{u} - |Dt||1-3z^2|\mathrm{Ci}(|Dt||1-3z^2|u)\right] \tag{S59}$$

We can pull $|Dt|$ out of the integral and replace $|Dt|u \to x$. We get

$$S(t) = \frac{4\pi}{3} Dt \lim_{x\to 0} \int_0^1 \mathrm{d}z \left[\frac{\sin((1-3z^2)x)}{x} - (1-3z^2)\mathrm{Ci}\left(|1-3z^2|x\right)\right] \tag{S60}$$

The divergence of the integrand is due to the cosine integral function. To deal with this, we rewrite it using

$$\mathrm{Ci}(\xi) = \gamma + \ln(\xi) + \mathrm{Cin}(\xi) \tag{S61}$$

where $\gamma$ is Euler's constant and $\mathrm{Cin}(\cdot)$ is the modified cosine integral function which satisfies $\mathrm{Cin}(0) = 0$. The full integral is now

$$S(t) = \frac{4\pi}{3} Dt \lim_{x\to 0} \int_0^1 \mathrm{d}z \left[\frac{\sin((1-3z^2)x)}{x} - (1-3z^2)\left[\gamma + \ln|1-3z^2| + \ln x + \mathrm{Cin}(|1-3z^2|x)\right]\right] \tag{S62}$$

The only term in the integrand that diverges for $x \to 0$ is $(1-3z^2)\ln x$. However, the $z$ integral over this term is zero, since the $z$ integral over $(1-3z^2)$ is zero. Therefore, we can drop the $\ln x$ term from the integrand. The divergent term along $x$ disappears due to symmetry along $z$! A similar argument allows us to drop the $(1-3z^2)\gamma$ term as well. All remaining terms in the integrand are finite everywhere and converge for $x \to 0$, so we can swap the $x$ limit and the $z$ integral. (In more technical

terms, the integrand converges uniformly over the entire $z$ interval, and we can apply the Lebesgue dominated convergence theorem to interchange the $x$ limit and the $z$ integral.)

$$S(t) = \frac{4\pi}{3} Dt \int_0^1 \mathrm{d}z \lim_{x\to 0} \left[ \frac{\sin((1-3z^2)x)}{x} - (1-3z^2)\ln|1-3z^2| - (1-3z^2)\mathrm{Cin}(|1-3z^2|x) \right] \tag{S63}$$

For $x = 0$, the first term becomes $(1-3z^2)$, which integrates to zero. The third term equals zero for $x = 0$ (since $\mathrm{Cin}(0) = 0$). We are left with

$$S(t) = \frac{4\pi}{3} Dt \int_0^1 \mathrm{d}z\, (3z^2-1)\ln|1-3z^2| \tag{S64}$$

Evaluation of this with Mathematica yields

$$S(t) = \frac{8\pi}{27}\left[3 + \sqrt{3}\ln(2-\sqrt{3})\right] Dt \tag{S65}$$

# S3 Numerical calculations

## S3.1 Monte Carlo simulation of intermolecular signal

To verify the analytical expression of the polarized background signal in Eq. (S42) and to investigate how it depends on neighboring spins, we performed Monte Carlo simulations. We start with Eq. (S30), and use an average over a finite number $N_\mathrm{conf}$ of configurations and a product over a finite number $N_\mathrm{B}$ of B-spins:

$$V_\mathrm{inter}(t_j) \approx \frac{1}{N_\mathrm{conf}} \sum_{c=1}^{N_\mathrm{conf}} \prod_{b=1}^{N_\mathrm{B}} V_\mathrm{AB}(\boldsymbol{r}_b, t_j) \tag{S66}$$

with the analytical signal Eq. (S29) for $V_\mathrm{AB}(\boldsymbol{r}_b, t_j)$. $t_j$ is a set of time points over which $V_\mathrm{inter}$ is evaluated.

The input parameters to this Monte Carlo model are the polarization $\epsilon$, the pump efficiency $p_\mathrm{B}$, the B-spin concentration $c_\mathrm{B}$, the number $N_\mathrm{B}$ of B-spins per configuration, and the number $N_\mathrm{conf}$ of configurations. For each configuration, an A-spin is placed at the origin of a spherical volume of radius $R = (3N_\mathrm{B}/4\pi c_\mathrm{B})^{1/3}$, and $N_\mathrm{B}$ B-spins are placed at random uniformly distributed positions $\boldsymbol{r}_b$ within the sphere using

$$\boldsymbol{r}_b = R \frac{u_r^{1/3}}{\sqrt{n_x^2 + n_y^2 + n_z^2}} \begin{pmatrix} n_x \\ n_y \\ n_z \end{pmatrix} \tag{S67}$$

where $u_r$ is drawn from the standard uniform distribution $\mathcal{U}(0,1)$, and $n_x$, $n_y$, and $n_z$ are drawn from the standard normal distribution $\mathcal{N}(0,1)$.

Figure S1 shows Monte Carlo simulations as a function of the number of excited B-spins in a configuration. The signal predicted analytically is captured perfectly. These simulations provide interesting insight into B-spin contributions to the decay. Remarkably, it is sufficient to consider configurations with only a single *excited* B-spin ($p_\mathrm{B}N_\mathrm{B} = 1$) in order to accurately recapture the initial 20% drop of the signal from its value at time zero. Both in-phase and out-of-phase signals are close to converged with 5 excited B-spins ($p_\mathrm{B}N_\mathrm{B} = 5$). With 50 excited B-spins, the entire decay curve is visually indistinguishable from the analytical model. These simulations show that the decay is dominated by just a few *excited* B-spins that are closest to the A-spin.

## S3.2 Nearest-neighbor distance distribution

In a uniform three-dimensional distribution of spins with number concentration $c$, the distribution of nearest-neighbor distances $r_\mathrm{nn}$ is given by (Berberan Santos, 1985)

$$P(r_\mathrm{nn}) = 4\pi c\, r_\mathrm{nn}^2 \exp\left(-\frac{4}{3}\pi c\, r_\mathrm{nn}^3\right) \tag{S68}$$

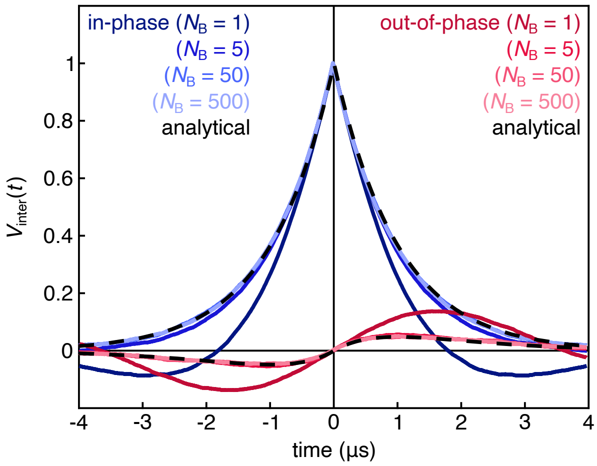


Figure S1: Monte Carlo model simulating the inter-molecular DEER signal $V_{\text{inter}}$ for maximum polarization ($\epsilon = 1$) and $p_B \cdot c_B = 1$ mM, assuming a sample with one A-spin and 75,000 configurations of a varying number $N_B$ of uniformly distributed B-spins. Changing $p_B \cdot c_B$ only affects the time scale. The analytical signals are shown as black dashed lines.

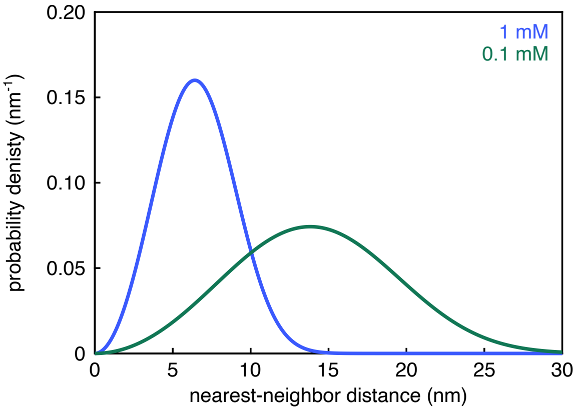


Figure S2: Nearest-neighbor distributions of spins in uniform three-dimensional distributions with overall concentrations 1 mM and 0.1 mM, based on Eq. (S68). The modes are at 6.4 and 13.8 nm, respectively.

The mode of this distribution is at $(2\pi c)^{-1/3} \approx 0.542\, c^{-1/3}$, and the mean is at $\Gamma(1/3)/(36\pi c)^{1/3} \approx 0.554\, c^{-1/3}$. The nearest-neighbor distance distributions for $c = 1$ mM and 0.1 mM are shown in Fig. S2.

## S4 Fit parameters

This section contains all of the fit parameters for the Q-band and G-band monoradical data (Table S1) and the G-band biradical data (Table S2) at various temperatures. Fitting was done using Eqs. (14)-(16), defined in the main text, with MATLAB's lsqcurvefit. All fit parameters are listed with the their 95% confidence intervals.

Table S1: The monoradical data were fit according to Eq. (14), where the fit parameters are the decay rate constant $k$, the overall signal amplitude $V_0$, the zero-time shift $t_0$, and an additional phenomenological fit factor $q_B$.

| Q-band | 11 K | | 50 K | |
|---|---|---|---|---|
| Parameter | Fit value | 95% confidence values | Fit value | 95% confidence values |
| $k$ | 0.2784 | 0.2773, 0.2794 | 0.1639 | 0.1633, 0.1646 |
| $V_0$ | 1.0064 | 1.0048, 1.0081 | 1.0051 | 1.0042, 1.0060 |
| $t_0$ | 3.4343 | 3.4308, 3.4378 | 2.4444 | 2.4414, 2.4474 |
| $q_B$ | 2.0587 | 1.8266, 2.2909 | 0.5769 | -0.2485, 1.4023 |
| G-band | 5 K | | 40 K | |
| Parameter | Fit value | 95% confidence values | Fit value | 95% confidence values |
| $k$ | 0.0632 | 0.0619, 0.0645 | 0.0566 | 0.0566, 0.0585 |
| $V_0$ | 1.0057 | 1.0030, 1.0085 | 1.0020 | 1.0000, 1.0041 |
| $t_0$ | 3.8749 | 3.8531, 3.8967 | 3.9496 | 3.9309, 3.9682 |
| $q_B$ | 3.5640 | 3.4294, 3.6985 | 0.7387 | 0.1355, 1.3419 |

Table S2: The biradical data were fit according to Eqs. (14)-(16), where the fit parameters are the inversion efficiency $p_B$, the decay rate constant $k$, overall signal amplitude $V_0$, the zero-time shift $t_0$, additional phenomenological fit factor for the intramolecular signal $q_F$ and intermolecular signal $q_B$, the peak position $r_0$ and the standard deviation $w$.

| G-band | 5 K | | 50 K | |
|---|---|---|---|---|
| Parameter | Fit value | 95% confidence values | Fit value | 95% confidence values |
| $p_B$ | 0.0328 | 0.0294, 0.0362 | 0.0329 | 0.0308, 0.0349 |
| $k$ | 0.0189 | 0.0181, 0.0196 | 0.0192 | 0.0187, 0.0196 |
| $V_0$ | 0.9987 | 0.9957, 1.0016 | 1.0016 | 0.9998, 1.0034 |
| $t_0$ | 0.4604 | 0.4406, 0.480 | 0.1648 | 0.1524, 0.1772 |
| $q_F$ | 1.0341 | 0.6547, 1.4135 | 0.0000 | -1.9133, 1.9133 |
| $q_B$ | 4.1766 | 3.8969, 4.4563 | 0.5342 | -0.4072, 1.4756 |
| $r_0$ | 3.7166 | 3.6603, 3.7728 | 3.8656 | 3.7892, 3.9420 |
| $w$ | 0.0897 | 0.0515, 0.1280 | 0.2198 | 0.1620, 0.2775 |

## S5 Experimental validation

This section contains the results of the various experiments conducted towards the aim of verifying the observed signal and resolving any discrepancies with the theoretical predictions. Among these experiments are pump–probe pulse excitation band overlap (Fig. S3), gain imbalance between the in-phase and out-of-phase detectors (Fig. S4), signals recorded on and off the echo to observe resonator background and phase instability (Fig. S5), and saturation recovery data (Fig. S7).

If there is a significant amount of pulse overlap, the pump pulse could partially excite A spins (and vice versa). This could lead to additional contributions to the DEER signal from secondary dipolar pathways (Fábregas-Ibáñez et al., 2022). The spectrum and pulse excitation profiles for the experimental conditions in the monoradical experiments (6.42 T, observer freuqency 180.000 GHz, pump frequency 180.050 GHz, pulse widths 58 ns) are shown in Fig. S3. Total excitation by the probe

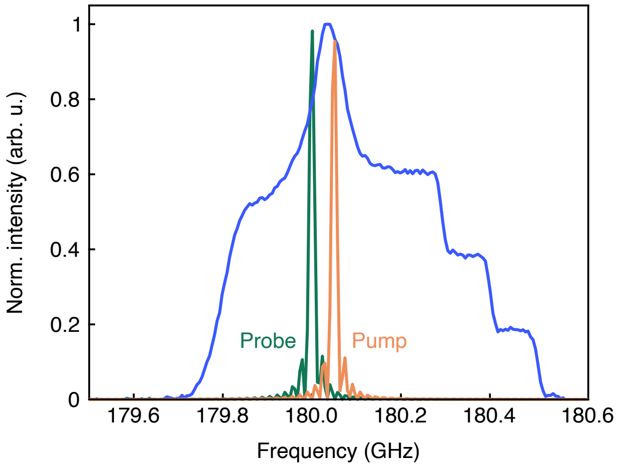


Figure S3: Frequency-swept spectrum (blue), converted from experimental field sweep, and excitation profiles for the pump (orange) and probe (green) pulses. The field sweep data was obtained for 1.0 mM solution of TEMPOL in 45:55 $D_2O$:$d_8$-glycerol at 50 K. Simulation parameters match those used in the monoradical experiments (observer frequency 180.000 GHz, pump frequency 180.050 GHz, pulse widths 36/58 ns, $\tau$ 500 ns, and repetition time 10 ms).

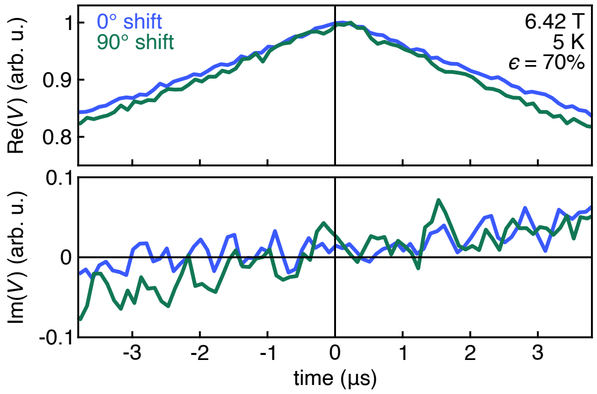


Figure S4: Experimental DEER traces for a 1.0 mM solution of TEMPOL in 45:55 $D_2O$:$d_8$-glycerol, measured at 180 GHz and 6.42 T at 5 K, run with no shift in detector phase (blue) and 90° shift (green). Experimental conditions are identical to those shown in the main text for the monoradical data at 6.42 T and 5 K.

pulse is approx. 3.7% and the pump pulse is approx. 4.3%. The overlap between pulse excitation profiles is approx. 2.7%. This is too small to create significant amplitudes in secondary dipolar pathways. Experiments with varying pump–probe offsets between 40 MHz and 120 MHz showed the same behavior (data not shown).

One possibility to get an overly intense out-of-phase component is that the second channel (out-of-phase with the echo) has a higher gain than the first channel. To test for gain imbalance, multiple traces of the monoradical data were recorded (Fig. S4). The first was run in the same manner as the experiments shown in the main text (blue), the second with the detector phase rotated by 90 degrees (green). Although not completely identical, there is no substantial difference in the relative amplitudes of the out-of-phase signals.

To verify that the observed out-of-phase signal is entirely due to the refocusing spins and not due to instrumental offsets, two traces of the monoradical sample were recorded, the first being run in the same manner as the experiments shown in the main text, the second recorded with the detector window offset in time from the echo (Fig. S5. The data show that there is no slope in the out-of-phase signal when recording off-echo, confirming that there is no instrumental offset or phase drift as a function of pump pulse position. The data also reveal that the actual out-of-phase signal is much noisier than the instrumental baseline obtained off-echo. This likely arises from some small phase

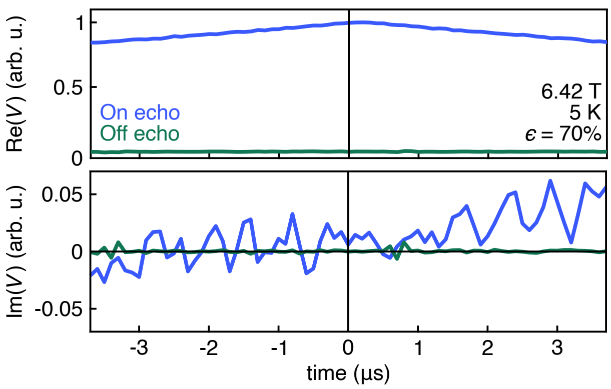


Figure S5: Experimental DEER traces for a 1.0 mM solution of TEMPOL in 45:55 $D_2O$:$d_8$-glycerol, measured at 180 GHz and 6.42 T at 5 K, run on echo (blue) and off echo (green). Experimental conditions are identical to those shown in the main text for the monoradical data at 6.42 T and 5 K.

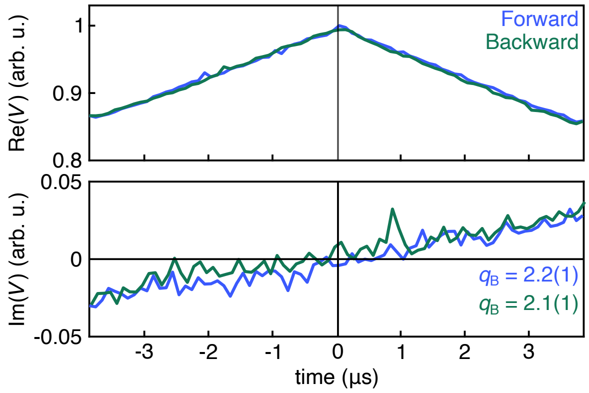


Figure S6: Experimental DEER traces for a 1.0 mM solution of TEMPOL in 45:55 $D_2O$:$d_8$-glycerol, measured at 180 GHz and 6.42 T at 5 K, run in the forward direction (blue) and the reverse direction (green). Experimental conditions are identical to those shown in the main text for the monoradical data at 6.42 T and 5 K. The data were fit with Eq. (14) and are shown with their 95% confidence interval in parentheses.

jitter in the instrument.

The detector phase drifts slowly over the (wall clock) course of the experiments. This could be a possible contributor to the observed mismatch between experiment and theory. However, it can be excluded because backwards and forward sweeps of $t$ give approximately the same result (Fig. S6), indicated by the identical fit factors (within 95% confidence). Regardless, the experiments were run with the shortest feasible acquisition times while still obtaining sufficient signal-to-noise to eliminate as much drift as possible.

To ensure that spin saturation was not occurring during experiments, a saturation recovery experiment was conducted to select the repetition time (Fig. S7). From the data shown, a 500 ms repetition time was used for all 5 K data shown.

The G-band setup typically utilizes a slower freezing method where the sample freezes after being placed inside the cold resonator. To ensure that the freezing procedure was not producing any unwanted effects on the experimental signal, i.e. aggregation upon freezing, two field sweeps (normalized) were obtained at 50 K for 250 μM biradical in $d_8$ toluene with different freezing procedures. The resulting spectra are shown in Fig. S8. The black trace shows the data for a sample that was frozen inside the resonator and the red trace shows data for a sample plunge frozen in liquid nitrogen outside the resonator. There is no significant difference.

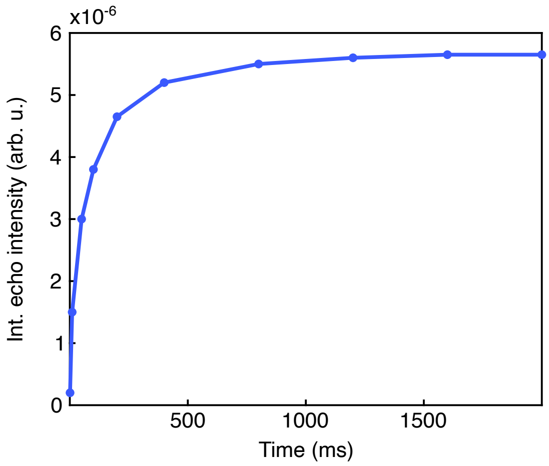


Figure S7: Saturation recovery experiment of a 1.0 mM solution of TEMPOL in 45:55 $D_2O$:$d_8$-glycerol sample, measured at 180 GHz and 6.42 T at 5 K.

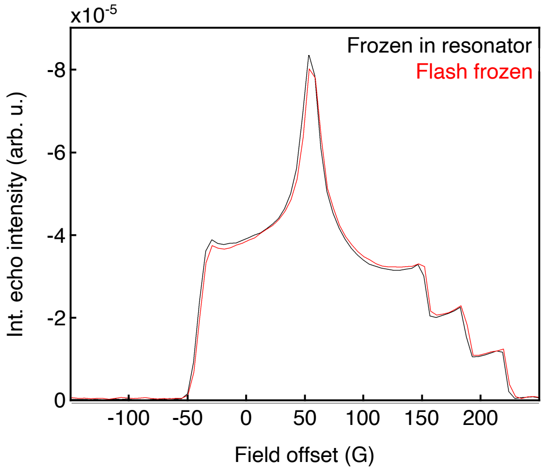


Figure S8: Field sweeps for a 0.25 mM solution of the biradical in deuterated toluene, measured at 180 GHz and 6.42 T at 50 K, for a sample frozen inside the resonator (black) and plunge frozen in liquid nitrogen (red).

# References

M. Berberan Santos. On the distribution of the nearest neighbor. *Am. J. Phys.*, 54:1139–1141, 1985. doi: https://doi.org/10.1119/1.14731.

L. Fábregas-Ibáñez, M. H. Tessmer, G. Jeschke, and S. Stoll. Dipolar pathways in dipolar EPR spectroscopy. *Phys. Chem. Chem. Phys.*, 24:2504–2520, 2022. doi: https://doi.org/10.1039/D1CP03305K.

A. Marko, V. Denysenkov, and T. F. Prisner. Out-of-phase PELDOR. *Mol. Phys.*, 111:2834–2844, 2013. doi: https://doi.org/10.1080/00268976.2013.807369.
